# Characterizing the urinary proteome of prematurity-associated lung disease in school-aged children

**DOI:** 10.1186/s12931-023-02494-3

**Published:** 2023-07-20

**Authors:** Christopher W Course, Philip A Lewis, Sarah J Kotecha, Michael Cousins, Kylie Hart, W John Watkins, Kate J Heesom, Sailesh Kotecha

**Affiliations:** 1 Department of Child Health, School of Medicine, Cardiff University, Heath Park, Cardiff, CF14 4XN UK; 2 Proteomics Facility, Faculty of Life Sciences, University of Bristol, Bristol, UK; 3 Department of Paediatrics, Cardiff and Vale University Health Board, Cardiff, UK

**Keywords:** Prematurity, Spirometry, Proteomics, Mass spectrometry, Inflammation, Lymphocytes

## Abstract

**Introduction:**

Although different phenotypes of lung disease after preterm birth have recently been described, the underlying mechanisms associated with each phenotype are poorly understood. We, therefore, compared the urinary proteome for different spirometry phenotypes in preterm-born children with preterm- and term-born controls.

**Methods:**

Preterm and term-born children aged 7–12 years, from the Respiratory Health Outcomes in Neonates (RHiNO) cohort, underwent spirometry and urine collection. Urine was analysed by Nano-LC Mass-Spectrometry with Tandem-Mass Tag labelling. The preterm-born children were classified into phenotypes of prematurity-associated preserved ratio impaired spirometry (pPRISm, FEV_1_ < lower limit of normal (LLN), FEV_1_/FVC ≥ LLN), prematurity-associated obstructive lung disease (POLD, FEV_1_ < LLN, FEV_1_/FVC < LLN) and preterm controls (FEV_1_ ≥ LLN,). Biological relationships between significantly altered protein abundances were analysed using Ingenuity Pathways Analysis software, and receiver operator characteristic curves were calculated.

**Results:**

Urine was analysed from 160 preterm-born children and 44 term controls. 27 and 21 were classified into the pPRISm and POLD groups, respectively. A total of 785 proteins were detected. Compared to preterm-born controls, sixteen significantly altered proteins in the pPRISm group were linked to six biological processes related to upregulation of inflammation and T-cell biology. In contrast, four significantly altered proteins in the POLD group were linked with neutrophil accumulation. Four proteins (DNASE1, PGLYRP1, B2M, SERPINA3) in combination had an area under the curve of 0.73 for pPRISm and three combined proteins (S100A8, MMP9 and CTSC) had AUC of 0.76 for POLD.

**Conclusions:**

In this exploratory study, we demonstrate differential associations of the urinary proteome with pPRISm and POLD.

**Trial registration:**

EudraCT: 2015-003712-20

**Supplementary Information:**

The online version contains supplementary material available at 10.1186/s12931-023-02494-3.

## Introduction

Preterm-born children, including those who developed the neonatal lung disease bronchopulmonary dysplasia (BPD, also known as chronic lung disease of prematurity), are at risk of low lung function (prematurity-associated lung disease, PLD) in childhood and beyond. Our recent systematic review reported > 9% difference in percent predicted forced expiratory volume in 1s (FEV_1_) between all preterm-born and term-born subjects in later life [1], increasing to 16% in those who had BPD in infancy. However, in multivariable regression models, gestational age at birth and intrauterine growth restriction (IUGR) are better predictors of PLD in childhood than BPD [2]. Furthermore, we have recently demonstrated different spirometry PLD phenotypes of prematurity-associated obstructive lung disease (POLD), prematurity-associated preserved ratio-impaired spirometry (pPRISm) and dysanapsis [3]. There is also concern that PLD is associated with early onset of chronic obstructive pulmonary disease (COPD) [4].

Historically, mechanistic studies have focussed on those with BPD in infancy, with evidence of smooth muscle extension into the distal airways in post-mortem samples from infants [5], and peri-bronchial fibrosis and CD8+ T-lymphocyte epithelial infiltrate in adolescent [6] and adult [7] survivors of BPD. A proportion of those with PLD will respond to inhaled therapies [8], however, a clearer understanding of the biological pathways underlying these PLD-associated phenotypes will aid their identification and development of targeted therapy. As urine lacks the same homeostatic mechanisms as blood, systemic protein changes accumulate and the urinary proteome may show alterations prior to clinical manifestations or histopathological changes to the lung tissue, reflecting earlier stages of disease development [9]. Urine proteomics has been used to study adult respiratory diseases [10], as well as BPD [11] and respiratory infections [12] in preterm-born infants, with the advantage that it can be sampled easily and non-invasively. The urinary proteome of neonates who develop BPD has shown increases of proteins associated with leukocyte mediated immunity, but with downregulation of myeloid cell lines and neutrophil degranulation [11], whereas those with infectious vs. non-infectious respiratory disease show differences in proteins related to cell adhesion, enzymatic regulation and inflammatory response [12]. However, to our knowledge, the changes in the urinary proteome in preterm-born individuals with lung function impairment in childhood has yet to be studied. We, therefore, performed exploratory analyses of the urinary proteome in preterm-born, school-aged children, with term-born matched controls, to elucidate the biological mechanisms underlying different PLD phenotypes of pPRISm and POLD.

## Methods

### Participants

This study was conducted on a cohort of children recruited to the Respiratory Health Outcomes in Neonates study (RHiNO, EudraCT: 2015-003712-20) which has been described extensively previously [2, 3, 8]. Briefly, children from a previous study [13] were supplemented with additional preterm-born children sourced from NHS Wales healthcare records and sent a respiratory and neurodevelopmental questionnaire if they were born ≤ 34 or ≥ 37 weeks’ gestation and were aged 7–12 years. Children with significant congenital malformations, cardiopulmonary or neuromuscular disease were excluded. Ethical approval was obtained from the South-West Bristol Research Ethics Committee (15/SW/0289). Parents gave informed written consent and children provided assent. Recruitment took place prospectively between November 2016 and September 2019.

Responders underwent spirometry (Microloop, Care Fusion, UK), performed according to ATS/ERS guidelines [14] and results were normalised using Global Lung Initiative (GLI) references [15] by trained research nurses. Any respiratory medications were withheld prior to their assessment (short- and long-acting β2-agonists for 8- and 48-hours respectively; inhaled corticosteroids for 24 h; and leukotriene receptor antagonists for 48 h) and children were free of respiratory infections for at least three weeks prior to testing. Low lung function in preterm-born children (PLD) was defined as FEV_1_ less than the lower limit of normal (LLN) as per GLI references [15]. Those with PLD were further categorised, as previously described [3], into pPRISm (FEV_1_ < LLN and FEV_1_/FVC ≥ LLN), and POLD groups (FEV_1_ < LLN with an FEV_1_/FVC < LLN). Preterm-born control (PT_c_) and term-born children had FEV_1_ ≥ LLN. BPD was defined as oxygen-dependency of 28-days or greater for those born < 32 weeks’ gestation and at 56 days of age for those born ≥ 32 weeks’ gestation [16]. Intrauterine growth restriction (IUGR) defined as birthweight < 10th percentile adjusted for sex and gestation (LMSgrowth v2.77, Medical Research Council, UK). Neonatal history was corroborated with medical records.

### Sample collection and analysis

Urine samples were obtained at the time of spirometry, aliquoted and stored at -80 °C on the day of collection until analysis.

### TMT labelling

Urine samples were analysed at the University of Bristol Proteomics Facility. 190 μl of urine was digested with trypsin (1.25 μg trypsin; 37 °C, overnight), labelled with Tandem Mass Tag (TMT) eleven plex reagents according to the manufacturer’s protocol (Thermo Fisher Scientific, Loughborough, UK) and the labelled samples pooled. The pooled sample was desalted using a SepPak cartridge according to the manufacturer’s instructions (Waters, Milford, Massachusetts, USA). Eluate from the SepPak cartridge was evaporated to dryness and resuspended in 1% formic acid prior to analysis by nano-LC MSMS using an Orbitrap Fusion Lumos mass spectrometer (Thermo Scientific).

### Nano-LC mass spectrometry

The TMT-labelled pool was fractionated using an Ultimate 3000 nano-LC system in line with an Orbitrap Fusion Lumos mass spectrometer (Thermo Scientific). In brief, peptides in 1% (vol/vol) formic acid were injected onto an Acclaim PepMap C18 nano-trap column (Thermo Scientific). After washing with 0.5% (vol/vol) acetonitrile 0.1% (vol/vol) formic acid peptides were resolved on a 250 mm × 75 μm Acclaim PepMap C18 reverse phase analytical column (Thermo Scientific) over a 150 min organic gradient, using 7 gradient segments (1–6% solvent B over 1 min, 6–15% B over 58 min, 15–32% B over 58 min, 32–40% B over 5 min, 40–90% B over 1 min, held at 90% B for 6 min and then reduced to 1% B over 1 min) with a flow rate of 300 nl min^− 1^. The TMT-labelled pool underwent a further fractionation to try and maximise peptide yield. The second fractionation used the above methodology again with a different gradient protocol: 6 gradient segments (1–6% solvent B over 1 min, 6–25% B over 118 min, 25–40%B over 3 min, 40–90%B over 1 min, held at 90%B for 6 min and then reduced to 1%B over 1 min.) again with a flow rate of 300 nl min^− 1^. Solvent A was 0.1% formic acid and Solvent B was aqueous 80% acetonitrile in 0.1% formic acid for both fractionation processes. Peptides were ionized by nano-electrospray ionization at 2.0 kV using a stainless-steel emitter with an internal diameter of 30 μm (Thermo Scientific) and a capillary temperature of 300 °C.

All spectra were acquired using an Orbitrap Fusion Lumos mass spectrometer controlled by Xcalibur 3.0 software (Thermo Scientific) and operated in data-dependent acquisition mode using an SPS-MS3 workflow. FTMS1 spectra were collected at a resolution of 120,000, with an automatic gain control (AGC) target of 200,000 and a maximum injection time of 50ms. Precursors were filtered with an intensity threshold of 5000, according to charge state (to include charge states 2–7) and with monoisotopic peak determination set to Peptide. Previously interrogated precursors were excluded using a dynamic window (60s ± 10ppm). The MS2 precursors were isolated with a quadrupole isolation window of 0.7 m/z. ITMS2 spectra were collected with an AGC target of 10,000, maximum injection time of 70ms and CID collision energy of 35%.

For FTMS3 analysis, the Orbitrap was operated at 50,000 resolution with an AGC target of 50,000 and a maximum injection time of 105ms. Precursors were fragmented by high energy collision dissociation (HCD) at a normalised collision energy of 60% to ensure maximal TMT reporter ion yield. Synchronous Precursor Selection (SPS) was enabled to include up to 5 MS2 fragment ions in the FTMS3 scan. All mass spectrometry runs were performed consecutively on the mass spectrometer with blank runs in between to prevent carry over from one experiment to the next.

### Data analysis

The raw data files were processed and quantified using Proteome Discoverer software v2.1 (Thermo Scientific) and searched against the UniProt Human database (downloaded October 2019: 150,786 entries) using the SEQUEST HT algorithm. Peptide precursor mass tolerance was set at 10ppm, and MS/MS tolerance was set at 0.6Da. Search criteria included oxidation of methionine (+ 15.995Da), acetylation of the protein N-terminus (+ 42.011Da) and Methionine loss plus acetylation of the protein N-terminus (-89.03Da) as variable modifications and carbamidomethylation of cysteine (+ 57.021Da) and the addition of the TMT mass tag (+ 229.163Da) to peptide N-termini and lysine as fixed modifications. Searches were performed with full tryptic digestion and a maximum of two missed cleavages were allowed. The reverse database search option was enabled, and all data was filtered to satisfy false discovery rate (FDR) of 5%. A more moderate stringency was applied to reduce the risk of important biological discoveries being classified as false negatives and allowed further consideration of the biological relevance of the protein through statistical and enrichment analysis.

### Statistical analysis

Baseline population characteristics were compared using Chi-squared or t-test as appropriate. Replicate numbers, i.e., number of samples in which a particular protein was detected, were calculated. To account for any dilutional effect on a urine sample altering total protein load, protein content was normalised using a central tendency method to the median protein abundance of its respective MS run, as previously described [11, 17]. Relative protein abundances, determined from the quantity of TMT-tag counts at each detected peptides spectral peak, between MS runs were scaled using pool samples. Samples with a total protein abundance ± 2-fold difference from the median MS run protein abundance were excluded to ensure accurate comparative quantitation by Proteome Discoverer v2.1.

Scaled protein abundances were log_2_-transformed and fold changes (log_2_FC) between groups were compared using Welch’s t-test. p < 0.05 was considered statistically significant. All analyses were performed using R v4.0.4 (R Foundation for Statistical Computing, Austria). Gene names synonymous with protein names have been used. Functional enrichment analysis (identifying changes in classes of proteins present) was performed with Webgestalt [18]. Ingenuity Pathways Analysis (IPA, Qiagen®, Germany) software identified functional relationships between significantly different protein abundances between groups, highlighting altered biological processes, and calculated activation z-scores, which predict biological process activation/inhibition based on published literature-derived gene-biological function relationships, with z-scores ± 2 considered significant. Receiver Operator Characteristic (ROC) curves were generated for biologically related proteins (identified by IPA), with high replicate numbers, between study groups to assess potential biomarker performance (assessed by area under the curve (AUC)), using two linear models, one based on the whole cohort and one using a leave-one-out cross validation (LOOCV) method. These proteins of interest were also analysed in univariable linear regression models to ascertain associations between these proteins and other early and current life factors. Associations with a p-value < 0.1 were combined into a multivariable model to examine the overall combined influence of each association.

## Results

### Participants

From 768 children (including 565 preterm-born and 203 term-born) recruited to RHiNO, urine samples were analysed from 270 participants. 64 (23.7%) samples were excluded as outliers (sample total protein abundance ± 2-fold difference from median protein abundance of respective MS run, as described above) (Additional file Fig. 1). Demographics of the included 206 participants are shown in Table 1. The demographics were largely similar between the included and excluded participants except rates of asthma were significantly higher and BPD were lower in included POLD group (Additional file Table 1). Preterm-born children were marginally older than the term-born children (mean ± SD 10.4 ± 1.4 years vs. 9.9 ± 1.1, p = 0.02) and had higher rates of asthma (39 (24.2%) vs. 2 (4.3%), p = < 0.001). 47 (29.2%) of the preterm-born subjects had received a neonatal diagnosis of mild/moderate/severe BPD, and 48 (30%) had an FEV_1_ < LLN. Of those, 27 (56%) were classified as pPRISm and 21 (44%) as POLD.

**Table 1:** Sample Demographics

Variable	Term born ($\ge$37/40) n = 46	Preterm born ($\le$34/40) n = 160	Preterm born Controls n = 112	pPRISm n = 27	POLD n = 21
Sex (male), n(%)	23 (50.0)	76 (47.5)	52 (46.4)	17 (63)	7 (33.3)
Ethnicity (white), n(%)	45 (97.8)	152 (95)	105 (93.8)	27 (100)	20 (95.2)
Age at testing (years), mean (SD)	9.9 (1.1)	10.4 (1.4)*	10.4 (1.3)	10.6 (1.6)	10.1 (1.7)
Weight (kg), mean (SD)	36.3 (10.3)	35.9 (9.7)	36.4 (9.5)	35.5 (9.1)	33.9 (11.8)
Body Mass Index (kg/m^2^), mean (SD)	18.1 (3.2)	17.5 (3.1)	17.7 (3.0)	17.0 (2.9)	17.0 (3.9)
Wheeze-ever, n(%)	12 (26.1)	97 (56.9)***	64 (57.1)	15 (55.6)	18 (85.7)†^#^
Doctor-diagnosed asthma, n(%)	2 (4.3)	39 (24.4)**	21 (18.8)	7 (25.9)	11 (52.4)††
*Neonatal Characteristics*					
Gestational age (weeks), mean (SD)	40.1 (1.2)	30.5 (2.8)***	30.4 (2.9)	30.9 (2.9)	30.2 (2.5)
Birthweight (g), mean (SD)	3499 (576)	1549 (594)***	1577 (607)	1587 (543)	1352 (572)
Birthweight (z-score), mean (SD)	0.1 (0.99)	0.11 (1.37)	0.3 (1.42)	-0.12 (0.9)	-0.45 (1.46)
Intrauterine growth restriction, n(%)	3 (6.5)	25 (15.6)	15 (13.4)	2 (7.4)	8 (38.1)††^##^
Antenatal Steroids, n(%)	0 (0)	137 (85.6)***	99 (88.4)	22 (81.5)	16 (76.2)
Mechanical ventilation, n(%)	0 (0)	70 (43.8)***	53 (47.3)	7 (25.9)	10 (47.6)
Bronchopulmonary Dysplasia (BPD), n(%)	0 (0)	47 (29.2)***	34 (30.4)	7 (25.9)	6 (28.6)
Antenatal smoking, n(%)	3 (6.5)	22 (13.8)	16 (14.3)	4 (14.8)	2 (9.5)

Preterm vs. Term: *p < 0.05, **p < 0.01, ***p < 0.001. pPRISm/POLD vs. Preterm born control: †p < 0.05, ††p < 0.01, †††p < 0.001. pPRISm vs. POLD: ^#^p < 0.05, ^##^p < 0.01

pPRISm: Prematurity-related preserved ratio with impaired spirometry. POLD: Prematurity-related obstructive lung disease. BPD: Bronchopulmonary dysplasia.

### Detected urinary proteins

A total of 785 proteins were detected, 735 (93.6%) of which were mapped to published gene names. 129 proteins were common to all samples. Functional enrichment analysis [18] was possible for 681 (86.8%) of the detected proteins (Additional file Fig. 2). 288 proteins were significantly different between any of the phenotype comparisons, and functional enrichment analysis was possible for 255 (88.5%). Overall, an enrichment of proteins related to metabolic processes, hydrolase activity and extracellular space/cell membrane activities was observed in the preterm-born groups.

### Comparison between the pPRISm group with preterm- and term-control groups

**Table 2:** Significantly altered biological processes in pPRISm & POLD vs. PT_c_ identified by IPA software

Gene Name	Protein Name	Protein Function	Protein ID FDR	Replicates	Log_2_FC	p-value	Inflammation of body cavity	Apoptosis myeloid cells	Quantity of leucocytes	Quantity of T-lymphocytes	Quantity of CD4 + T-lymphocytes	Quantity of CD8 + T-lymphocytes	Accumulation of neutrophils
pPRISm vs. PT_c_ (n = 27 vs. 112)													
GLA	Alpha-galactosidase A	Lipid metabolism	0.002	2 v 6	-1.06	0.0001			●				
CLEC11A	C-type lectin domain family 11 member A	Osteogenesis	0.01	3 v 15	-1.39	0.001			●				
NAGLU	Alpha-N-acetylglucosaminidase	Glycosidase/ hydrolase	< 0.001	25 v 107	-0.34	0.011	●						
PGLYRP1	Peptidoglycan recognition protein 1	Innate immunity	< 0.001	27 v 112	-0.40	0.012	●		●	●	●		
DNASE1	Deoxyribonuclease-1	Serum endonuclease	< 0.001	27 v 112	-0.53	0.013	●		●	●	●	●	
MYH9	Myosin-9	Cell adhesion/shape	< 0.001	4 v 8	0.49	0.014	●						
SERPINA3	Alpha-1-antichymotrypsin	Serine protease inhibitor	< 0.001	25 v 100	-0.36	0.014	●	●					
CTSV	Cathepsin L2	Thiol protease	0.035	16 v 73	-0.33	0.015	●		●	●	●		
AGT	Angiotensinogen (Serpin A8)	Regulation of blood pressure	< 0.001	4 v 28	0.81	0.019	●		●	●		●	
ANXA1	Annexin A1	Inflammatory/immune response	< 0.001	7 v 29	1.46	0.026	●	●	●	●	●	●	
CLEC4G	C-type lectin domain family 4 member G	Substrate binder	< 0.001	27 v 106	-0.36	0.028	●		●	●	●	●	
SCGB1A1	Uteroglobin	Phospholipase A2 inhibitor	< 0.001	5 v 33	-1.04	0.032	●		●				
MGAM	Maltase-glucoamylase	Carbohydrate metabolism	< 0.001	27 v 112	-0.35	0.038	●						
B2M	Beta-2-microglobulin	Class I major histocompatibility complex	< 0.001	27 v 112	-0.60	0.040	●		●	●		●	
ANPEP	Aminopeptidase N	Aminopeptidase	< 0.001	23 v 103	-0.37	0.040		●	●	●	●		
CD14	Monocyte differentiation antigen CD14	Inflammatory/immune response	< 0.001	27 v 112	-0.39	0.045	●	●					
**POLD vs. PT**_**c**_ **(n = 21 vs. 112)**													
MMP9	Matrix metalloproteinase-9	Collagen degradation, leukocyte migration	< 0.001	21 v 105	0.30	0.018							●
AGT	Angiotensinogen (Serpin A8)	Regulation of blood pressure	< 0.001	5 v 28	0.55	0.030							●
S100A8	Protein S100-A8	Inflammatory/immune response	< 0.001	21 v 107	0.69	0.034							●
CTSC	Dipeptidyl peptidase 1 (Cathepsin C)	Thiol protease	< 0.001	19 v 77	0.31	0.038							●

Protein ID FDR: Protein identification false discovery rate; measure of confidence for the correct identification of the protein. Log_2_FC: log2 fold change of protein abundances between groups. p values given by Welch’s t-test

**Fig. 1:**
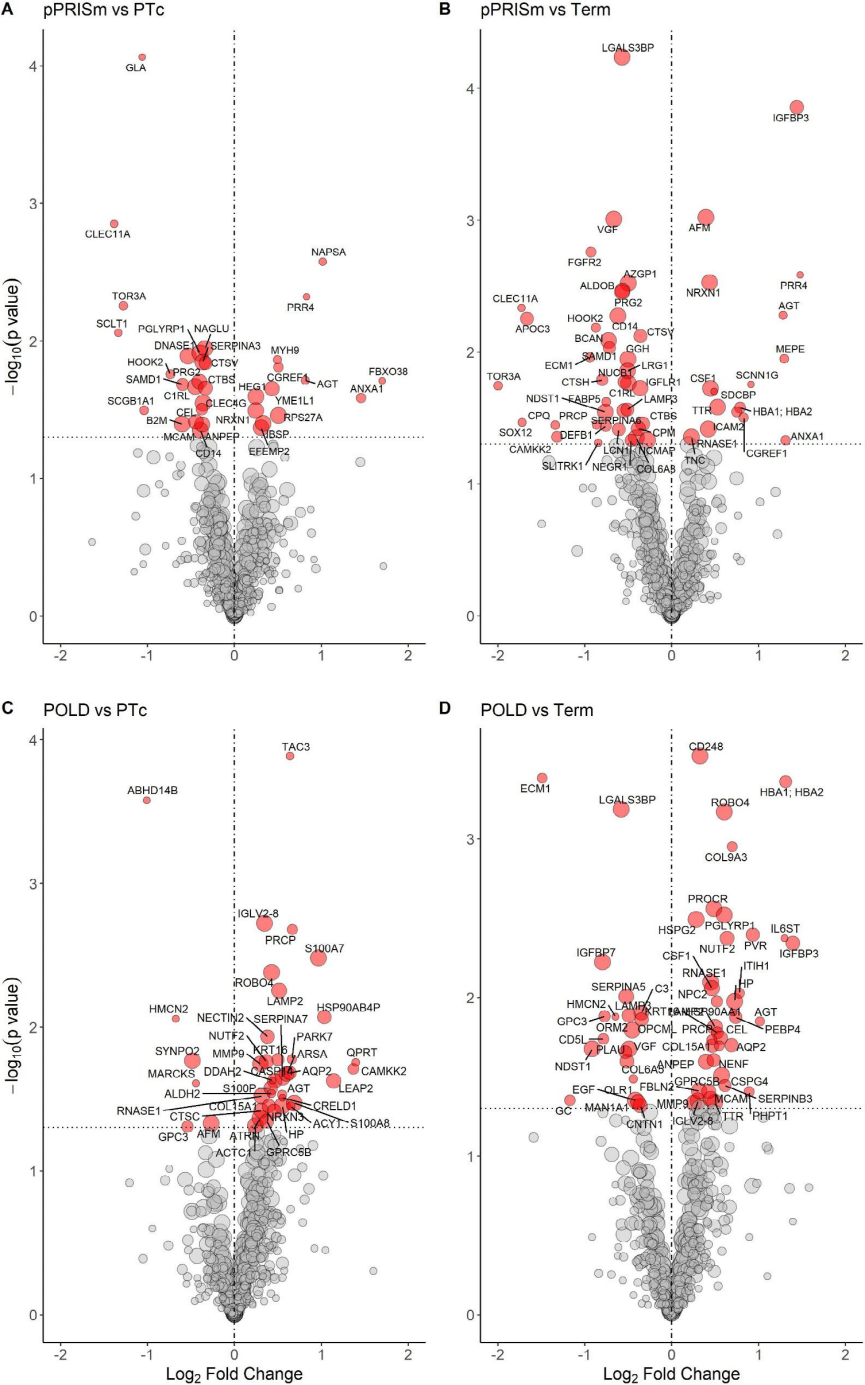
Volcano Plots showing significant protein differences for pPRISm and POLD phenotypes compared to PT_c_ and Term groups. Vertical line represents Log_2_ fold change of 0. Horizontal line equivalent to a p-value of 0.05. Proteins with a significant difference between groups highlighted and labelled with respective gene name. Size of circle relative to replicate number. POLD: Prematurity-related obstructive lung disease. pPRISm: Prematurity-related preserved ratio with impaired spirometry. PT_c_: Preterm-born controls

**Fig. 2:**
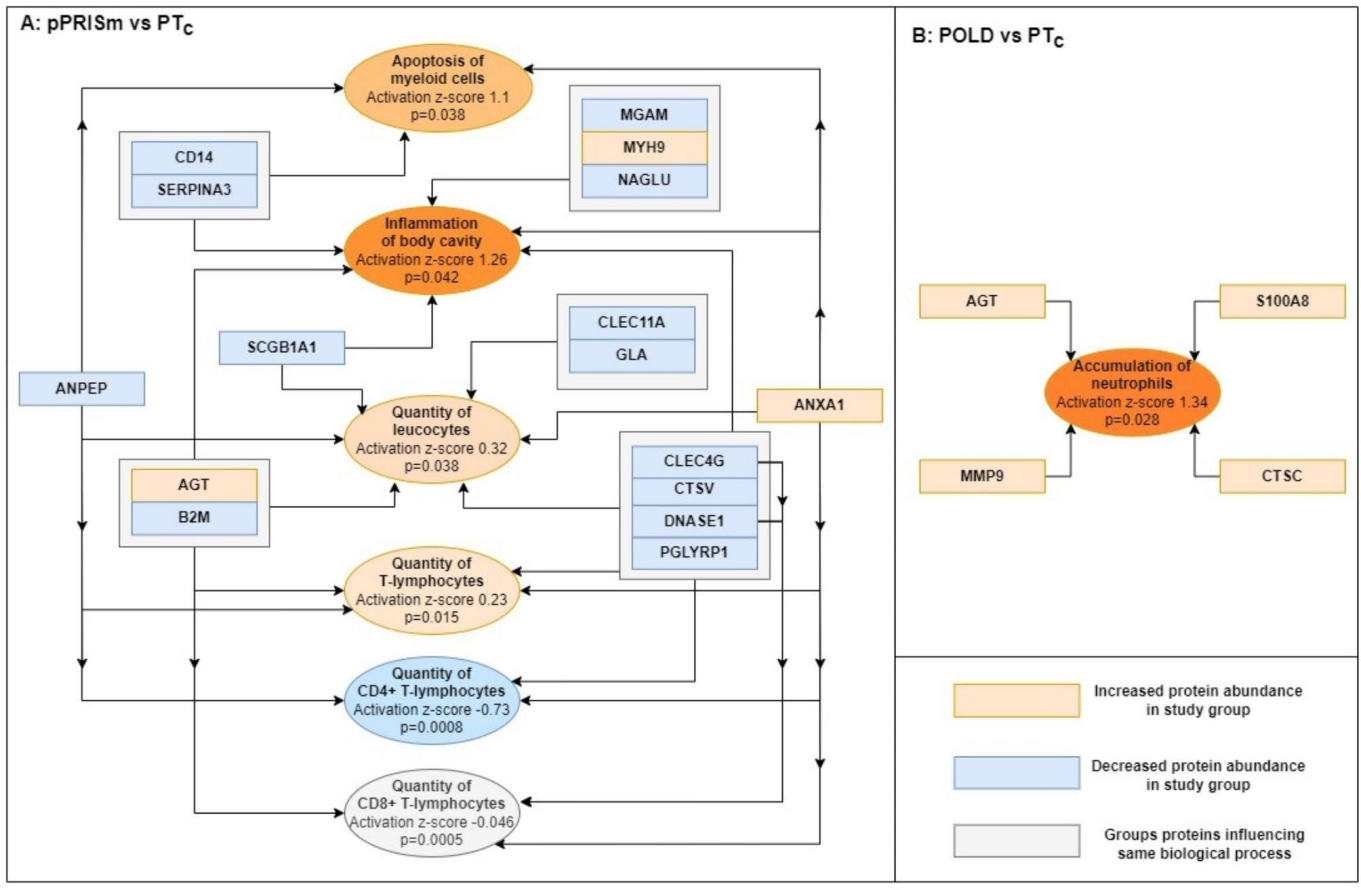
Proteins linked with significantly altered biological processes by IPA software within lung function phenotypes (pPRISm and POLD compared to PT_c_). POLD: Prematurity-related obstructive lung disease. pPRISm: Prematurity-related preserved ratio with impaired spirometry. PT_c_: Preterm-born controls

37 (5.3%) proteins had significantly different abundance when compared to PT_c_ (Fig. 1; Additional file Table 2), and 62 (8.9%) when compared to the term-born group (Fig. 1; Additional file Table 3). 14 proteins were common between the two comparisons. IPA linked 16 significantly altered proteins in pPRISm compared to PT_c_ to six biological processes (Table 2; Fig. 2); *Inflammation of body cavity* (PGLYRP1, DNASE1, MYH9, SERPINA3, CTSV, AGT, ANXA1, CLEC4G, SCGB1A1, B2M, CD14) (p = 0.042); *Apoptosis of myeloid cells* (SERPINA3, ANXA1, ANPEP, CD14) (p = 0.038); *Quantity of leucocytes* (GLA, CLEC11A, PGLYRP1, DNASE1, CTSV, AGT, ANXA1, CLEC4G, SCGB1A1, B2M, ANPEP) (p = 0.038); and *Quantity of T-lymphocytes* (PGLYRP1, DNASE1, CTSV, AGT, ANXA1, CLEC4G, B2M, ANPEP) (p = 0.015). IPA-calculated activation z-scores suggested upregulation of these processes (Fig. 2). There was also a significant link between these proteins and the quantities of CD4+ (p = 0.008) and CD8+ (p = 0.005) T-lymphocytes, with a suggestion of a downregulation of CD4 + T-lymphocytes (activation z-score − 0.73). IPA analysis of significantly different protein abundances in pPRISm group compared to the Term-born group linked six proteins (AGT, CD14, CSF1, FABP5, HBB, ANXA1) with *Synthesis of prostaglandin* (p = 0.038, activation z-score 1.23)). Five proteins (PRG2, MGAM, CD14, LGALS3BP, ANXA1) were significantly linked with neutrophil activation (p = 0.038, z-score − 0.64).

**Fig. 3:**
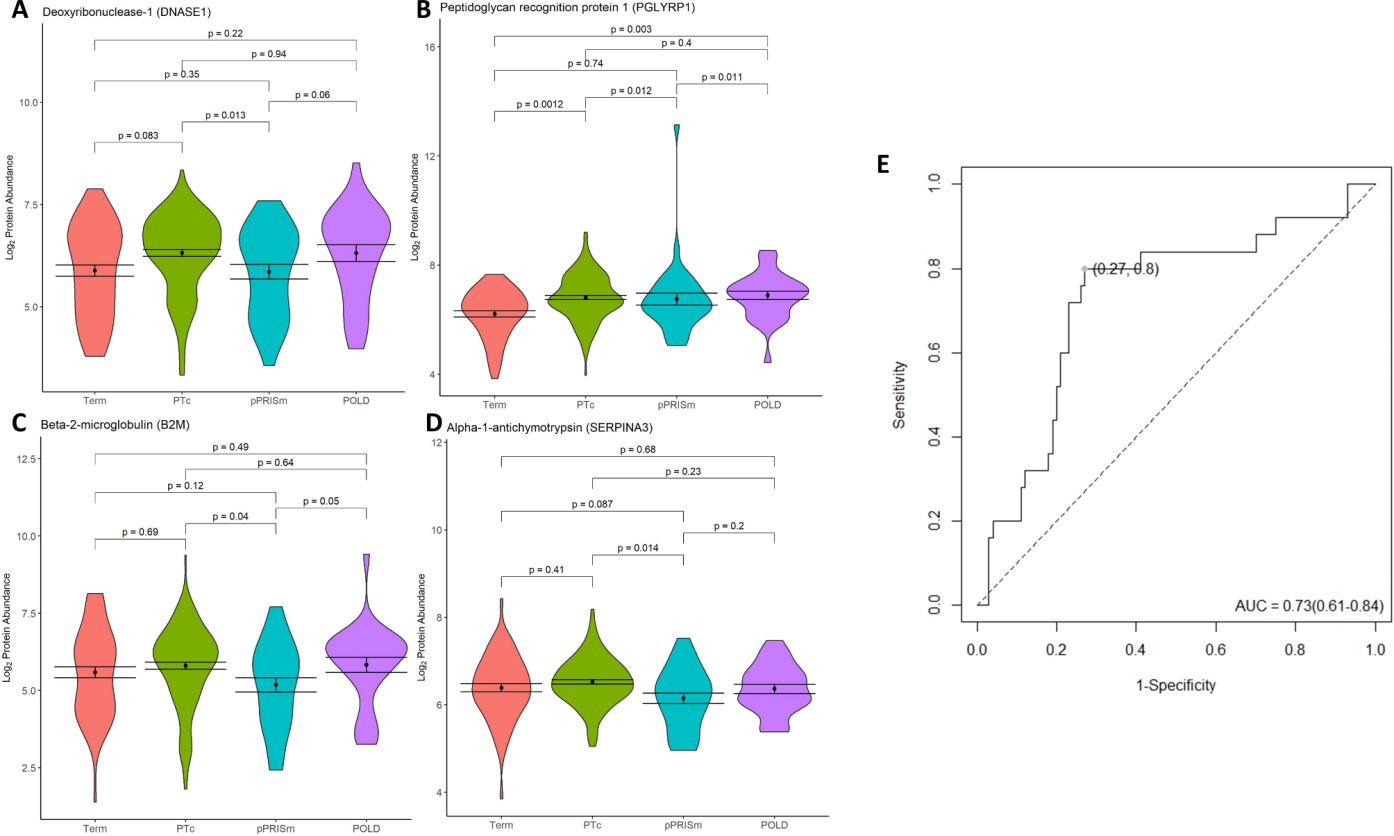
Significantly altered protein abundances in pPRISm vs. PT_c_ comparisons, showing violin plots for (**A**) DNASE1, (**B**) PGLYRP1, (**C**) B2M, and (**D**) SERPINA3, including comparisons with pPRISm and Term groups. (**E**) ROC Curve analysis for DNASE1, PGLYRP1, B2M and SERPINA3 in combination for pPRISm vs. PT_c_. Youden point given. For violin plots, black dot represents mean, bars standard error of the mean. p-values given for between group comparisons. POLD: Prematurity-related obstructive lung disease. pPRISm: Prematurity-related preserved ratio with impaired spirometry. PT_c_: Preterm-born controls. AUC: Area under the curve

ROC analysis (Table 3; Fig. 3) demonstrated that DNASE1, PGLYRP1, B2M and SERPINA3 in combination had the highest predictive ability for identifying pPRISm from within the preterm group (AUC: 0.73 (95% confidence interval 0.61, 0.84), sensitivity 0.80 (0.64, 0.96), specificity 0.73 (0.64, 0.82), p = < 0.001). Using the LOOCV model, the predictive ability of this protein panel was AUC 0.65 (0.52, 0.78), p = 0.01 (Additional file Table 6; Additional file Fig. 3). Results from univariable and multivariable linear regression modelling for these proteins are shown in Table 4. DNASE1, PGLYRP1, B2M remained significantly associated with pPRISm in multivariable modelling (p-values 0.008, 0.011, 0.018 respectively) with B2M also being significantly associated with a history of BPD in the multivariable model (p = 0.003). No other life factors were significantly associated with SERPINA3 on univariable models, with pPRISm being highly significant (p = 0.005).

**Table 3:** ROC Analysis of high replicate proteins implicated in related biological functions by IPA software. POLD: Prematurity-related obstructive lung disease. pPRISm: Prematurity-related preserved ratio with impaired spirometry. PT_c_: Preterm-born controls. CI: Confidence Interval. PPV: Positive predictive value. NPV: Negative predictive value

Protein(s) included in model	Replicates	AUC (95% CI)	p-value	Sensitivity (95% CI)	Specificity (95% CI)	PPV (95% CI)	NPV (95% CI)
**pPRISm vs. PT**_c_ **(n = 27 v 112)**							
DNASE1	27 v 112	0.66 (0.55, 0.78)	0.004	0.56 (0.37, 0.74)	0.72 (0.64, 0.80)	0.33 (0.19, 0.46)	0.87 (0.80, 0.94)
PGLYRP1	27 v 112	0.64 (0.53, 0.75)	0.012	0.93 (0.83, 1.00)	0.32 (0.23, 0.40)	0.25 (0.16, 0.33)	0.95 (0.87, 1.02)
B2M	27 v 112	0.63 (0.51, 0.75)	0.020	0.59 (0.41, 0.78)	0.66 (0.60, 0.75)	0.30 (0.18, 0.42)	0.87 (0.80, 0.94)
SERPINA3	25 v 100	0.66 (0.53, 0.79)	0.007	0.48 (0.28, 0.68)	0.82 (0.75, 0.90)	0.40 (0.23, 0.58)	0.86 (0.79, 0.93)
DNASE1 + PGLYRP1 + B2M + SERPINA3	25 v 100	0.73 (0.61, 0.84)	< 0.001	0.80 (0.64, 0.96)	0.73 (0.64, 0.82)	0.43 (0.28, 0.57)	0.94 (0.88, 0.99)
**POLD vs. PT**_c_ **(n = 21 v 112)**							
S100A8	21 v 107	0.64 (0.52, 0.76)	0.021	0.82 (0.66, 0.98)	0.50 (0.40, 0.59)	0.25 (0.15, 0.35)	0.93 (0.86, 1.00)
MMP9	21 v 105	0.64 (0.51, 0.77)	0.023	0.48 (0.26, 0.69)	0.76 (0.68, 0.84)	0.29 (0.14, 0.44)	0.88 (0.81, 0.95)
CTSC	19 v 77	0.66 (0.53, 0.79)	0.015	0.68 (0.48, 0.89)	0.65 (0.54, 0.76)	0.33 (0.18, 0.47)	0.89 (0.81, 0.97)
S100A8 + MMP9 + CTSC	19 v 77	0.76 (0.63, 0.90)	< 0.001	0.84 (0.68, 1.00)	0.61 (0.50, 0.72)	0.35 (0.21, 0.49)	0.94 (0.87, 1.00)

**Table 4:** Univariable and multivariable linear regression analysis of early and current life factors and proteins of interest in pPRISm compared to PT_c_

Variable	DNASE1			PGLYRP1			B2M			SERPINA3		
	Beta	SE	p-value	Beta	SE	p-value	Beta	SE	p-value			
Univariable Models												
Sex, ref = Male	0.14	0.17	0.39	0.21	0.13	0.11	0.14	0.22	0.52	0.13	0.10	0.22
Age at testing, years	-0.07	0.06	0.23	0.05	0.05	0.32	-0.08	0.08	0.30	0.03	0.04	0.44
IUGR ref = No IUGR	-0.11	0.26	0.68	-0.34	0.21	*0.09*	-0.28	0.34	0.41	0.15	0.17	0.40
BPD ref = No BPD	-0.32	0.18	*0.08*	-0.24	0.14	0.10	-0.67	0.23	0.005*	0.07	0.12	0.54
POLD ref = PT_c_	-0.53	0.21	0.011*	-0.40	0.16	0.016*	-0.60	0.27	0.027*	-0.37	0.13	0.005*
**Multivariable Models**												
IUGR ref = No IUGR	-	-	**-**	-0.38	0.20	*0.06*	-	-	-	Not taken forward for multivariable model		
BPD ref = No BPD	-0.33	0.18	*0.06*	-	-	-	-0.69	0.23	0.003*			
pPRISm ref = PT_c_	-0.55	0.20	0.008*	-0.42	0.16	0.011*	-0.63	0.26	0.018*			

SE: Standard error; IUGR: Intrauterine growth restriction; BPD: Bronchopulmonary dysplasia, pPRISm: prematurity-associated preserved ratio-impaired spirometry, PT_c_: preterm-born controls

Bold*: p value < 0.05 *Italic*: p value < 0.1. Dashes indicate variables that had p ≥ 0.1 on univariable analysis and therefore not included in multivariable model. Multivariable models only created where ≥ 2 univariable models had a p-value < 0.1

### Comparison between the POLD group with preterm- and term-control groups

The POLD group had several significant differences when compared with the PT_c_ group (Table 1) including increased wheeze-ever (85.7% vs. 57.1%, p = 0.027), asthma (52.4% vs. 18.8%, p = 0.001) and IUGR (38.1% vs. 13.4%, p = 0.006). When compared to the pPRISm group (Table 1), POLD had higher wheeze-ever (85.7% vs. 55.6%, p = 0.025) and higher rates of IUGR (38.1% vs. 7.4%, p = 0.009).

44 (6.4%) proteins had a significantly different abundance when compared to PT_c_ (Fig. 1; Additional file Table 4), and 70 (10.1%) when compared to term-born subjects (Fig. 1, Additional file Table 5) with 18 proteins being common within the two comparisons. IPA analyses linked four significantly altered proteins (AGT, CTSC, MMP9, S100A8) to *Accumulation of neutrophils* when the POLD and PT_c_ groups were compared (p = 0.028, z-score 1.34, Table 2; Fig. 2). IPA linked eight significantly altered proteins with *Cellular infiltration by macrophages* (AGT, PLAU, C3, MMP9, CSF1, PROCR, IL6ST, PRCP) when the POLD and Term-born groups were compared (p = 0.011, activation z-score 0.59).

**Fig. 4:**
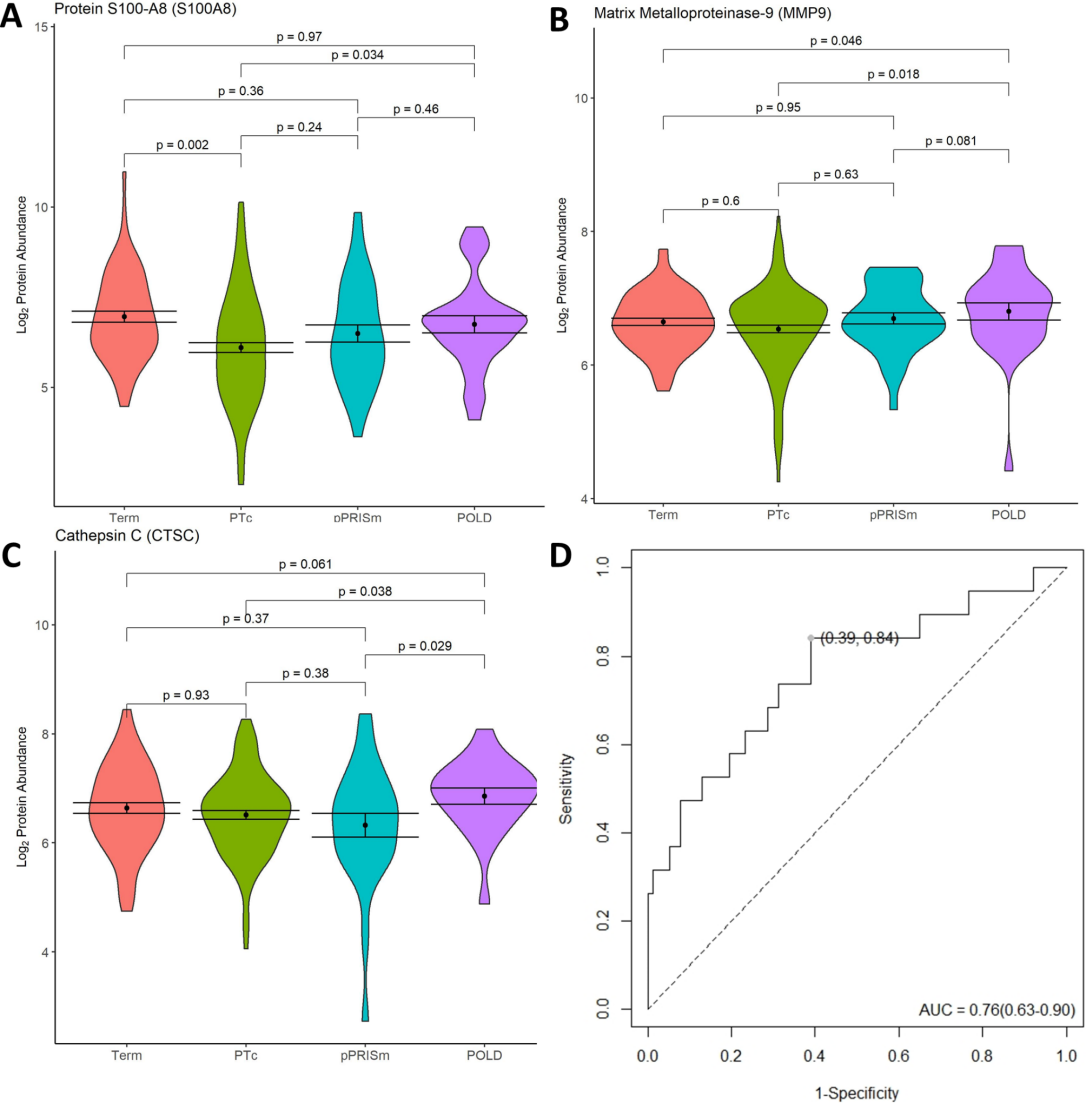
Significantly altered protein abundances in POLD vs. PT_c_ comparisons, showing violin plots for (**A**) S100A8, (**B**) MMP9 and (**C**) CTSC, including comparisons with pPRISm and Term groups. (**D**) ROC Curve analysis for S100A8, MMP9 and CTSC in combination for POLD vs. PT_c_. Youden point given. For violin plots, black dot represents mean, bars standard error of the mean. p-values given for between group comparisons. POLD: Prematurity-related obstructive lung disease. pPRISm: Prematurity-related preserved ratio with impaired spirometry. PT_c_: Preterm-born controls. AUC: Area under the curve

ROC analysis (Table 3; Fig. 4) demonstrated that S100A8, MMP9 and CTSC in combination had the highest predictive ability for identifying POLD from within the preterm group (AUC 0.76 (0.63–0.90), sensitivity 0.84 (0.68, 1.00), specificity 0.61 (0.50, 0.72), p = < 0.001). Using the LOOCV model, S100A8, MMP9 and CTSC in combination performed similarly (AUC 0.72 (0.57–0.86), p = 0.002) (Additional file Table 6; Additional file Fig. 3). Results from univariable and multivariable linear regression modelling for these proteins are given in Table 5. No other early or current life factors were significantly associated with S100A8 and CTSC abundance in univariable models. A history of BPD was significantly associated with MMP9 abundance in univariable modelling (p = 0.017) and remained significant in the multivariable model BPD (p = 0.017), along with POLD (p = 0.024).

**Table 5:** Univariable and multivariable linear regression analysis of early and current life factors and proteins of interest in POLD compared to PT_c_

Variable	MMP9			S100A8			CTSC		
	Beta	SE	p-value	Beta	SE	p-value	Beta	SE	p-value
Univariable Models									
Sex, ref = Male	0.15	0.10	0.15	-0.42	0.26	0.11	-0.02	0.13	0.88
Age at testing, years	-0.01	0.04	0.72	-0.04	0.09	0.65	0.01	0.05	0.78
IUGR ref = No IUGR	-0.02	0.13	0.90	0.32	0.34	0.36	-0.10	0.17	0.55
BPD ref = No BPD	-0.26	0.11	0.017*	0.38	0.28	0.17	-0.03	0.15	0.85
POLD ref = PT_c_	-0.30	0.13	0.025*	-0.69	0.34	0.043*	-0.31	0.16	*0.058*
**Multivariable Models**									
BPD ref = No BPD	-0.26	0.11	0.017*	Not taken forward for multivariable model			Not taken forward for multivariable model		
POLD ref = PT_c_	-0.30	0.13	0.024*						

SE: Standard error; IUGR: Intrauterine growth restriction; BPD: Bronchopulmonary dysplasia, POLD: prematurity-associated obstructive lung disease, PT_c_: preterm-born controls. Multivariable models only created where ≥ 2 univariable models had a p-vale < 0.1

Bold*: p value < 0.05 *Italic*: p value < 0.1

## Discussion

In this novel exploratory study, we have characterised the urinary proteome of two phenotypes of PLD, namely pPRISm and POLD, in a large cohort of preterm-born children. We have demonstrated increased abundance of proteins related to inflammatory processes and immune-system function in preterm-born children with low lung function, several years after the initial pulmonary insult occurred in the neonatal period. In those with a pPRISm phenotype, there was evidence of multiple affected biological processes, with ongoing inflammatory process occurring with suggested alteration in T-lymphocyte biology. In contrast, in the POLD group altered biological processes focusing on myeloid cell lines including neutrophil and macrophage activity appear to be affected.

It is apparent that there is greater complexity to PLD, with a need to understand the biological mechanisms or endotypes underlying the different phenotypes of lung disease to understand their pathogenesis. Such identification of endotypes will aid the development of specific targeted therapeutic interventions. For this reason, we have focussed our analysis using current lung function to define the different phenotypes rather than using historical diagnoses of BPD, or other associated life factors, that could influence current lung function. PRISm has recently been described in the adult population to be strongly associated with increased development of COPD, cardiovascular disease and all-cause mortality [19, 20]. We recently reported this specific phenotype in preterm-born children with a different association to bronchodilator response, fraction exhaled nitric oxide (FE_NO_) and early/current life factors compared to POLD and PT_c_ groups [3].

Our urinary proteomic analyses using these phenotypes revealed some interesting hypothesis-generating observations. There were multiple associations with systemic alterations in inflammatory and immune processes postulated in the pPRISm group, with a likely increase in inflammation, overall quantities of leucocytes and, in particular, T-lymphocytes. This observation has recently been corroborated by Um-Bergstrom et al. who reported relative decrease of CD4+ T-cells and increase of CD8+ T-cells in bronchoalveolar lavage fluid from young adults with former BPD, a similar finding to those with COPD. CD8+ T-cells were also negatively correlated with both FEV_1_ and FEV_1_/FVC [7]. Adolescent survivors of severe BPD have also been noted to have an increase in bronchial wall CD8 + lymphocytes [6]. A recent urine metabolomic study has linked early increases in proteins associated with leukocyte mediated immunity to the later development of BPD in infants born < 29 weeks’ gestation [11]. Our data also suggested that CD4+ lymphocytes may be downregulated in the pPRISm group, of which only 25.9% had a previous history of BPD. A relative increase in CD8+ T-cells number and function has also been associated with severity of COPD [21], and in adult subjects, PRISm is a known risk factor for development of COPD [22]. Four proteins (DNASE1, PGLYRP1, B2M and SERPINA3) showed good predictive ability for identifying pPRISm from PT_c_ in ROC analysis. Deoxyribonuclease-1 (DNASE1) is a ubiquitous endonuclease which degrades the majority of circulating free DNA released from apoptosis and necrotic cell death, with DNASE1 deficiency being previously reported to be associated with autoimmune disease in animal models and humans [23]. Peptidoglycan recognition protein 1 (PGLYRP1), an innate proinflammatory and antibacterial protein, has been linked with asthma in animal models, with PGLYRP1-deficient mice exhibiting a decreased Th2/CD4+ response, with a less severe phenotype [24]. Increased serum beta-2-microglobulin (B2M), the light chain of the class I major histocompatibility complex, has been linked with COPD disease progression, namely development of pulmonary fibrosis, alveolar wall thickening and decreased gas exchange capacity [25]. The anti-protease alpha-1-antichymotrypsin (SERPINA3) manipulates the immune and inflammatory response through inhibition of chymotrypsin and cathepsin G. Previous studies have identified increased SERPINA3 in serum from COPD subtypes associated with metabolic syndrome [26], with genetic mutations resulting in SERPINA3 deficiency resulting in milder disease in patients with COPD [27] and cystic fibrosis(CF) [28]. The reduced abundances, we observed, of these four proteins in pPRISm were all significantly linked with a possible upregulation of inflammatory processes, with DNASE1, PGLYRP1 and B2M also being significantly linked with T-cell biology.

IPA analyses of increased abundance of the four proteins (MMP9, AGT, S100A8, CTSC) in the POLD group (when compared to PT_c_) suggested increased neutrophil accumulation, which is a reasonable hypothesis given the association of neutrophilic inflammation with wheezing in asthma [29]. Whether this is a specific phenotype of PLD or has similarities with neutrophilic asthma will need further investigation. Matrix metalloproteinase-9 (MMP9) is a gelatinase protease, stored in neutrophils, involved in activating proinflammatory cytokines, enhancing inflammatory cell migration, and degradation of the extracellular matrix. Increased MMP9 in respiratory samples has been linked with several lung diseases, including paediatric patients with acute respiratory distress syndrome (ARDS) [30]. Increased MMP9 has also been observed in preterm-born neonates who subsequently develop BPD [31, 32], including a recent urine metabolomic study where early increase in MMP9 had a high predictive ability for development of BPD in extremely preterm infants [11]. MMP9 had a significant association with BPD in our cohort in univariable modelling, which remained in our multivariable regression model, along with a significant association with POLD. We have previously shown a significant association between BPD and the development of a POLD phenotype [3]. In older subjects, elevated serum MMP9 has been linked with COPD exacerbations [33] and FEV_1_ decline in CF [34]. Cathepsin C (CTSC) is a serine protease released by neutrophils that can result in increased tissue-degradation, being implicated in the pathophysiology of pneumonia and ARDS in mechanically ventilated adults [35]. S100A8 is also associated with acute lung injury, being secreted by degranulating neutrophils and bronchial epithelium during infection/inflammation [36]. It has been shown to be increased in lung diseases resulting in tissue remodelling, including in bronchiolitis obliterans in children [37], and in adults with CF and COPD [38]. These three proteins all have a role in tissue remodelling; we have recently reported that the POLD group has significantly altered ventilation mechanics on hyperpolarised ^129^Xe ventilation and diffusion MRI imaging [39], which is likely to be related to tissue remodelling. MMP9, S100A8 and CTSC in combination had good predictive ability for identifying the POLD group using ROC analysis. Whether these combinations of proteins have prospective predictive value for PLD phenotypes prior to the development of lung function deficits will require further work.

In this study, we have analysed the urinary proteome. Whilst this is not a lung-specific sample type, it is easily and non-invasively obtainable, and has previously been utilised in the study of respiratory diseases in neonates [11] and adults [10]. In addition, as urine lacks the same homeostatic controls as blood, proteome changes in urine may be detectable at an earlier stage of disease [9] which makes it an attractive sample type to study in preterm-born children, as they may be at a milder or pre-symptomatic stage of respiratory impairment, as their lung function continues to develop through adolescence into adulthood [40].

This study represents one of the largest proteomic analyses of urine in the paediatric population, and although lung dysfunction was present in approximately 30% of the preterm-born group, this is the first study to our knowledge that has examined the urinary proteome of this cohort. Our regression modelling has demonstrated that many of the protein changes we have seen are primarily related to current lung function phenotype. We have used a robust TMT-methodology to quantify protein abundances and allow accurate comparisons between phenotypes, however there may have been proteins with low abundances/low TMT-tag counts that did not reach the limit of detection of the mass spectrometer. To ensure accurate quantitation with Proteome Discoverer software we excluded a number of samples to ensure robust findings, however we saw minimal significant differences in the participant characteristics between included and excluded samples. Whilst our TMT-based methodology gives robust protein abundances for comparative purposes, it does not give absolute protein concentrations within a sample, which would need to be determined to directly apply this data clinically. Whilst our study lacks a validation cohort, we are limited by the number of available large cohorts of preterm-born children who experienced a contemporary standard of neonatal care from which to sample.

In conclusion, we have demonstrated distinct changes in the urinary proteome associated with the two recently described phenotypes of PLD; POLD and pPRISm. There was suggestion of proteins associated with the inflammatory and immune systems in the pPRISm group and of potential neutrophilic inflammation in the POLD group. We have also demonstrated potential predictive ability of combinations of proteins to identify the POLD and pPRISm phenotypes. Further work with specific targeting of these proteins is now required to confirm if these proteins can be used clinically to screen prospectively for preterm-born children at risk of future lung dysfunction, or whether they can be targeted therapeutically.

## Electronic supplementary material

Below is the link to the electronic supplementary material.

Additional file 1: Contains supplementary figures and tables to give further detail to the methods and results section

## Data Availability

The data generated and analysed that support the findings of this study are included in this published article [and its supplementary information file, Additional File 1.pdf]. The mass spectrometry proteomics data have been deposited to the ProteomeXchange Consortium via the PRIDE partner repository with the dataset identifier PXD042759. Further data from the RHiNO study are available to research collaborators subject to confidentiality and non-disclosure agreements. Contact Professor Sailesh Kotecha (kotechas@cardiff.ac.uk) for any data requests.
