# Supplementary material for: Characterizing the urinary proteome of prematurity-associated lung disease in school-aged children
Source: Respir Res. 2023 Jul 20;24:191. doi: 10.1186/s12931-023-02494-3 (PMC10357627; doi:10.1186/s12931-023-02494-3)

Characterizing the urinary proteome of prematurity-associated lung disease in school-aged children - Additional File:

Christopher W Course[1], Philip A Lewis[2], Sarah J Kotecha[1], Michael Cousins[1,3], Kylie Hart[3], W John Watkins[1], Kate J Heesom[2], Sailesh Kotecha[1].

[1]Department of Child Health, School of Medicine, Cardiff University, Cardiff, United Kingdom.

[2]Proteomics Facility, Faculty of Life Sciences, University of Bristol, Bristol, United Kingdom

[3]Department of Paediatrics, Cardiff and Vale University Health Board, Cardiff, United Kingdom

Corresponding Author: Professor Sailesh Kotecha

Department of Child Health

Cardiff University School of Medicine

Heath Park

Cardiff CF14 4XN

United Kingdom

kotechas@cardiff.ac.uk

Additional File Table 1: Comparing included and excluded samples: POLD: Prematurity-related obstructive lung disease. pPRISm: Prematurity-related preserved ratio with impaired spirometry.

Variable	Term born (≥37/40)		Preterm born (≤34/40)		Preterm born Controls		POLD		pPRISm	
	Included	Excluded	Included	Excluded	Included	Excluded	Included	Excluded	Included	Excluded
	n = 46	n = 20	n = 160	n = 44	n = 112	n = 28	n = 21	n = 6	n = 27	n = 10
Sex (male), n(%)	23 (50.0)	11 (55)	76 (47.5)	22 (50)	52 (46.4)	14 (50)	7 (33.3)	3 (50)	17 (63)	5 (50)
Ethnicity (white), n(%)	45 (97.8)	20 (100)	152 (95)	42 (95.5)	105 (93.8)	26 (92.9)	20 (95.2)	6 (100)	27 (100)	10 (100)
Age at testing (years), mean (SD)	9.9 (1.1)	10.3 (1.0)	10.4 (1.4)	10.4 (1.3)	10.4 (1.3)	10.0 (1.3)	10.1 (1.7)	10.9 (1.2)	10.6 (1.6)	11.3 (1.0)
Weight (kg), mean (SD)	36.3 (10.3)	36.1 (10.4)	35.9 (9.7)	37.2 (10.9)	36.4 (9.5)	36.7 (11.7)	33.9 (11.8)	36.0 (9.0)	35.5 (9.1)	39.2 (10.6)
Body Mass Index (kg/m^2), mean (SD)	18.1 (3.2)	17.3 (3.0)	17.5 (3.1)	18.4 (3.6)	17.7 (3.0)	18.6 (3.8)	17.0 (3.9)	17.7 (2.8)	17.0 (2.9)	18.2 (3.7)
Wheeze-ever, n(%)	12 (26.1)	5 (25)	97 (56.9)	22 (50)	64 (57.1)	16 (57.1)	18 (85.7)	3 (50)	15 (55.6)	3 (30)
Doctor-diagnosed asthma, n(%)	2 (4.3)	1 (5)	39 (24.4)	5 (11.4)	21 (18.8)	3 (10.7)	**11 (52.4)**	**0 (0)***	7 (25.9)	2 (20)
Neonatal Characteristics										
Gestational age (weeks), mean (SD)	40.1 (1.2)	40.4 (1.1)	30.5 (2.8)	30.5 (3.0)	30.4 (2.9)	30.8 (3.1)	30.2 (2.5)	28.6 (2.7)	30.9 (2.9)	31.0 (2.5)
Birthweight (g), mean (SD)	3499 (576)	3650 (627)	1549 (594)	1575 (650)	1577 (607)	1642 (672)	1352 (572)	1050 (428)	1587 (543)	1702 (590)
Birthweight (z-score), mean (SD)	0.1 (0.99)	0.2 (1.2)	0.11 (1.37)	0.15 (1.52)	0.3 (1.42)	0.3 (1.6)	-0.45 (1.46)	-0.82 (1.20)	-0.12 (0.9)	0.3 (1.4)
Intrauterine growth restriction, n(%)	3 (6.5)	2 (10)	25 (15.6)	7 (15.9)	15 (13.4)	3 (10.7)	8 (38.1)	3 (50)	2 (7.4)	1 (10)
Antenatal Steroids, n(%)	0 (0)	0 (0)	137 (85.6)	39 (88.6)	99 (88.4)	24 (85.7)	16 (76.2)	6 (100)	22 (81.5)	9 (90)
Mechanical ventilation, n(%)	0 (0)	0 (0)	70 (43.8)	18 (40.9)	53 (47.3)	11 (39.3)	10 (47.6)	5 (83.3)	7 (25.9)	2 (20)
BPD, n(%)	0 (0)	0 (0)	47 (29.2)	14 (31.8)	34 (30.4)	7 (25)	**6 (28.6)**	**5 (83.3)***	7 (25.9)	2 (20)
Antenatal smoking, n(%)	3 (6.5)	0 (0)	22 (13.8)	5 (1.4)	16 (14.3)	4 (14.3)	2 (9.5)	0 (0)	4 (14.8)	1 (10)

Independent samples T-test/Chi-squared test; Included vs Excluded: *p<0.05, **p<0.01, ***p<0.001

POLD: Prematurity-related obstructive lung disease. pPRISm: Prematurity-related preserved ratio with impaired spirometry. BPD: Bronchopulmonary dysplasia. BDR: bronchodilator response. FE_{NO}: fraction exhaled nitric oxide.

Additional File Figure 1: Log_2 total protein abundance of each sample separated by mass spectrometry (MS) run (given in number above each graph). Solid red line represents median of total protein abundance of each MS run, with dashed lines representing ±2-fold difference of MS run median total protein abundance. Samples with a total protein abundance >±2-fold difference from median of total protein abundance of the MS run highlighted in red as outliers and excluded from further analysis.

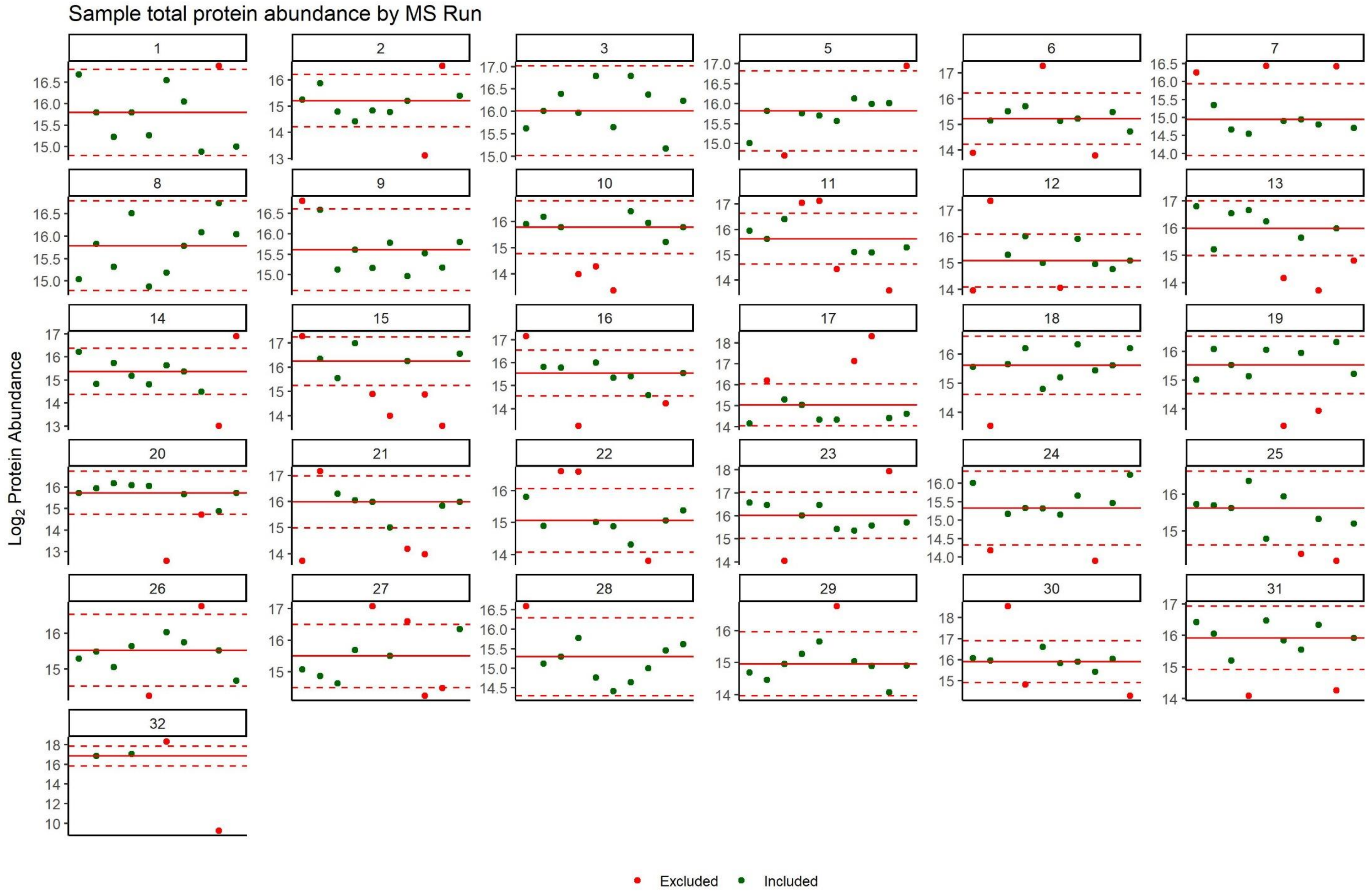

Additional File Figure 2: Functional enrichment analysis of detected urine proteome. Black bar on each column represents the expected background enrichment level.

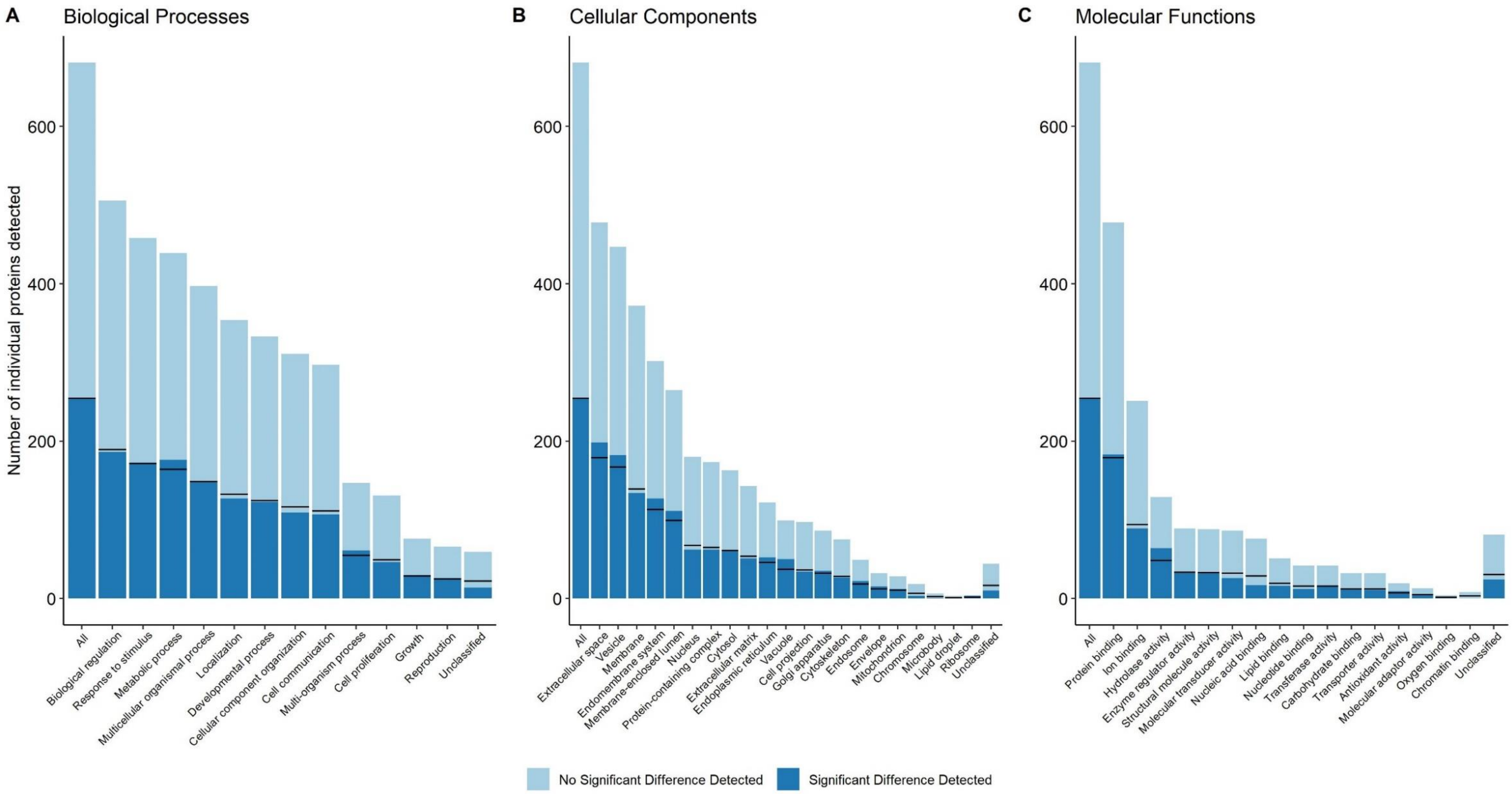

Additional file Table 2: Significantly altered protein abundances between pPRISm vs PTc.

UniProt Accession	Gene Name	Protein Name	Protein Function	pPRISm vs PTc (n = 27 v 112)		
				Replicates	Log_2FC	p
P06280	GLA	Alpha-galactosidase A	Lipid metabolism	2 v 6	-1.06	0.0001
Q9Y240	CLEC11A	C-type lectin domain family 11 member A	Osteogenesis	3 v 15	-1.39	0.001
A0A2U8J8Y8	IgH	Ig heavy chain variable region	Immune response	2 v 6	-1.86	0.002
O96009	NAPSA	Napsin-A	May be involved in processing of pneumocyte surfactant precursors.	3 v 17	1.01	0.003
Q16378	PRR4	Proline-rich protein 4	Extracellular space/Visual perception	2 v 15	0.83	0.005
Q9H497	TOR3A	Torsin-3A	Nucleotide binding	5 v 17	-1.28	0.006
Q96NL6	SCLT1	Sodium channel and clathrin linker 1	Anchors basal body to plasma membrane	3 v 11	-1.34	0.009
P54802	NAGLU	Alpha-N-acetylglucosaminidase	Glycosidase/hydrolase	25 v 107	-0.34	0.011
O75594	PGLYRP1	Peptidoglycan recognition protein 1	Innate immunity	27 v 112	-0.40	0.012
P24855	DNASE1	Deoxyribonuclease-1	Serum endonuclease	27 v 112	-0.53	0.013
P35579	MYH9	Myosin-9	Cell adhesion/cell shape	4 v 8	0.49	0.014
P01011	SERPINA3	Alpha-1-antichymotrypsin	Serine protease inhibitor	25 v 100	-0.36	0.014
O60911	CTSV	Cathepsin L2	Thiol protease	16 v 73	-0.33	0.015
Q99674	CGREF1	Cell growth regulator with EF hand domain protein 1	Cell adhesion, cell cycle	7 v 22	0.51	0.016
Q96ED9	HOOK2	Protein Hook homolog 2	Protein transport	6 v 24	-0.74	0.017
P01019	AGT	Angiotensinogen (Serpin A8)	Regulation of blood pressure	4 v 28	0.81	0.019
Q6PIJ6	FBXO38	F-box only protein 38	Adaptive immune response	2 v 11	1.70	0.020
P13727	PRG2	Bone marrow proteoglycan	Immune response, antimicrobial	22 v 91	-0.41	0.020
Q6SPF0	SAMD1	Sterile alpha motif domain-containing protein 1	Chromatin regulator	13 v 63	-0.60	0.021
Q9NZP8	C1RL	Complement C1r subcomponent-like protein	Serine protease, complement pathway	25 v 105	-0.45	0.021
Q01459	CTBS	Di-N-acetylchitobiase	Glycosidase	19 v 87	-0.34	0.022
Q96TA2	YME1L1	ATP-dependent zinc metalloprotease YME1L1	Metalloprotease	21 v 80	0.43	0.022
Q9ULI3	HEG1	Protein HEG homolog 1	Developmental protein, heart and vessel formation	27 v 111	0.25	0.025
P04083	ANXA1	Annexin A1	Inflammatory/immune response	7 v 29	1.46	0.026
Q6UXB4	CLEC4G	C-type lectin domain family 4 member G	Substrate binder, cell receptor for virus entry	27 v 106	-0.36	0.028
P19835	CEL	Bile salt-activated lipase	Serine esterase, lipid degredation	12 v 64	-0.38	0.031
B1AHL2	FBLN1	Fibulin-1	Cell adhesion/migration	4 v 24	0.49	0.031
P11684	SCGB1A1	Uteroglobin	Phospholipase A2 inhibitor	5 v 33	-1.04	0.032
P58400	NRXN1	Neurexin-1-beta	Cell adhesion	26 v 107	0.24	0.032
P62979	RPS27A	Ubiquitin-40S ribosomal protein S27a	Ribonucleoprotein	27 v 111	0.51	0.035
A0A0J9YVZ3	MGAM	Maltase-glucoamylase, intestinal	Carbohydrate metabolism	27 v 112	-0.35	0.038
P43121	MCAM	Cell surface glycoprotein MUC18	Cell adhesion	21 v 87	-0.45	0.039
P21815	IBSP	Bone sialoprotein 2	Biomineralization, cell adhesion	26 v 101	0.33	0.040
P61769	B2M	Beta-2-microglobulin	Class I major histocompatibility complex	27 v 112	-0.60	0.040
P15144	ANPEP	Aminopeptidase N	Aminopeptidase	23 v 103	-0.37	0.040
O95967	EFEMP2	EGF-containing fibulin-like extracellular matrix protein 2	Elastic fibre formation	25 v 101	0.30	0.043
P08571	CD14	Monocyte differentiation antigen CD14	Immune and inflammatory response	27 v 112	-0.39	0.045

Additional file Table 3: : Significantly altered protein abundances between pPRISm vs Term-born children

UniProt Accession	Gene Name	Protein Name	Protein Function	pPRISm vs Term (n = 27 v 46)		
				Replicates	Log_2FC	p
Q08380	LGALS3BP	Galectin-3-binding protein	Cell adhesion	27 v 46	-0.57	0.0001
P17936	IGFBP3	Insulin-like growth factor-binding protein 3	Growth regulation	18 v 31	1.44	0.0001
P43652	AFM	Afamin	Protein transport	27 v 46	0.39	0.001
A0A2U8J953	IgH	Ig heavy chain variable region (Fragment)	Immune response	9 v 7	-2.45	0.001
O15240	VGF	Neurosecretory protein VGF	Neurogenesis/neuroplasticity	27 v 46	-0.67	0.001
P21802	FGFR2	Fibroblast growth factor receptor 2	Cell proliferation regulator	8 v 8	-0.93	0.002
Q16378	PRR4	Proline-rich protein 4	Extracellular space/Visual perception	2 v 7	1.48	0.003
P58400	NRXN1	Neurexin-1-beta	Cell adhesion	26 v 46	0.44	0.003
Q9UL78	Unknown	Myosin-reactive immunoglobulin light chain variable region (Fragment)	Unknown	22 v 46	-0.44	0.003
P25311	AZGP1	Zinc-alpha-2-glycoprotein	Lipid degradation	27 v 46	-0.50	0.003
P05062	ALDOB	Fructose-bisphosphate aldolase B	Glycolysis	23 v 46	-0.57	0.003
P13727	PRG2	Proteoglycan 2	Immune response, antimicrobial	22 v 38	-0.57	0.004
Q9Y240	CLEC11A	C-type lectin domain family 11 member	Osteogenesis	3 v 9	-1.73	0.005
P01019	AGT	Angiotensinogen (Serpin A8)	Regulation of blood pressure	4 v 8	1.28	0.005
P08571	CD14	Monocyte differentiation antigen CD14	Immune and inflammatory response	27 v 46	-0.62	0.005
P02656	APOC3	Apolipoprotein C	Lipid metabolism	16 v 26	-1.67	0.006
Q96ED9	HOOK2	Protein Hook homolog 2	Protein transport	6 v 16	-0.87	0.007
O60911	CTSV	Cathepsin L2	Thiol protease	16 v 39	-0.36	0.008
Q96GW7	BCAN	Brevican core protein	Nervous system development	26 v 34	-0.73	0.008
Q8TE24	MGAM	Maltase-glucoamylase	Carbohydrate metabolism	5 v 7	-1.31	0.008
Q6SPF0	SAMD1	Sterile alpha motif domain-containing protein 1	Chromatin regulator	13 v 28	-0.72	0.009
Q99985	SEMA3C	Semaphorin-3C	Developmental protein	6 v 14	2.72	0.010
Q16610	ECM1	Extracellular matrix protein 1	Multifunctional, protease binding	6 v 15	-0.94	0.011
Q9NQ76	MEPE	Matrix extracellular phosphoglycoprotein	Post-translational protein phosphorylation	10 v 5	1.29	0.011
Q92820	GGH	Gamma-glutamyl hydrolase	Hydrolase	27 v 46	-0.50	0.011
Q4TZM4	HBB	Hemoglobin beta chain	Oxygen transport	5 v 7	1.47	0.013
P02750	LRG1	Leucine-rich alpha-2-glycoprotein	Extracellular protein	25 v 46	-0.51	0.014
Q8NEJ1	Unknown	Uncharacterized protein	Unknown	4 v 7	-0.87	0.016
P09668	CTSH	Pro-cathepsin H	Lysosomal protein degradation	11 v 15	-0.80	0.016
Q02818	NUCB1	Nucleobindin-1	Calcium/G-protein binding	16 v 39	-0.54	0.017
Q9NZP8	C1RL	Complement C1r subcomponent-like protein	Serine protease, complement pathway	25 v 45	-0.50	0.017
P51170	SCNN1G	Amiloride-sensitive sodium channel subunit gamma	Ion channel	2 v 9	0.91	0.018
Q9H497	TOR3A	Torsin-3A	Nucleotide binding	5 v 6	-2.00	0.018
A0A0X9UWL5	Unknown	GCT-A5 light chain variable region (Fragment)	Unknown	22 v 46	-0.41	0.019
P09603	CSF1	Macrophage colony-stimulating factor 1	Cytokine	26 v 39	0.45	0.019
Q9H665	IGFLR1	IGF-like family receptor 1	Cell membrane receptor IGF	24 v 34	-0.36	0.019
O00560	SDCBP	Syntenin-1	Multifunctional protein trafficker	2 v 24	0.49	0.020
Q01469	FABP5	Fatty acid-binding protein 5	Lipid metabolism	5 v 20	-0.75	0.024
P02766	TTR	Transthyretin	Thyroid hormone-binding protein	26 v 41	0.53	0.026

P69905	HBA1; HBA2	Hemoglobin subunit alpha	Oxygen transport	10 v 37	0.78	0.026
Q9UQV4	LAMP3	Lysosome-associated membrane glycoprotein 3	Adaptive immunity	16 v 34	-0.51	0.027
P08185	SERPINA6	Corticosteroid-binding globulin	Glucocorticoid/progestin transport	19 v 34	-0.55	0.028
P13598	ICAM2	Intercellular adhesion molecule 2	Leukocyte adhesion	8 v 18	0.75	0.028
P52848	NDST1	Bifunctional heparan sulfate N-deacetylase/N-sulfotransferase 1	Multifunctional enzyme/immune response	26 v 44	-0.76	0.029
Q99674	CGREF1	Cell growth regulator with EF hand domain protein 1	Cell adhesion, cell cycle	7 v 11	0.83	0.031
O15370	SOX12	Transcription factor SOX-12	Transcription factor	4 v 7	-1.72	0.034
Q01459	CTBS	Di-N-acetylchitobiase	Glycosidase	19 v 45	-0.34	0.035
Q9UL85	Unknown	Myosin-reactive immunoglobulin kappa chain variable region (Fragment)	Unknown	13 v 33	-1.01	0.035
P42785	PRCP	Lysosomal Pro-X carboxypeptidase	Carboxypeptidase	6 v 28	-0.86	0.036
Q9Y646	CPQ	Carboxypeptidase Q	Carboxypeptidase	5 v 7	-1.34	0.036
P60022	DEFB1	Beta-defensin 1	Bactericidal activity	11 v 20	-0.76	0.036
P14384	CPM	Carboxypeptidase M	Carboxypeptidase	20 v 30	-0.38	0.038
P07998	RNASE1	Ribonuclease pancreatic	Endonuclease	27 v 46	0.42	0.038
P31025	LCN1	Lipocalin-1	Transport protein	19 v 16	-0.61	0.039
Q96RR4	CAMKK2	Calcium/calmodulin-dependent protein kinase kinase 2	Serine/threonine-protein kinase	10 v 15	-1.32	0.044
P24821	TNC	Tenascin	Cell adhesion	27 v 46	0.23	0.044
P12111	COL6A3	Collagen alpha-3(VI) chain	Cell adhesion	27 v 46	-0.41	0.045
Q7Z3B1	NEGR1	Neuronal growth regulator 1	Cell adhesion	13 v 25	-0.47	0.047
Q5T1S8	NCMAP	Noncompact myelin-associated protein	Myelin formation	27 v 46	-0.28	0.047
P04083	ANXA1	Annexin A1	Inflammatory/immune response	7 v 20	1.31	0.047
Q96PX8	SLITRK1	SLIT and NTRK-like protein 1	Synaptogenesis	3 v 13	-0.85	0.049
S6BGE0	Unknown	IgG H chain	Immune response	8 v 20	0.44	0.049

Additional file Table 4: : Significantly altered protein abundances between POLD vs PTc

UniProt Accession	Gene Name	Protein Name	Protein Function	POLD vs PTc (n = 21 v 112)		
				Replicates	Log_2FC	p
B2R582	Unknown	cDNA, FLJ92374, highly similar to CLEC3B, mRNA	Unknown	2 v 12	-1.59	0.000003
Q9UHF0	TAC3	Tachykinin-3	Neuropeptide	3 v 22	0.64	0.0001
Q96IU4	ABHD14B	Protein ABHD14B	Acyltransferase	2 v 11	-1.01	0.0003
C9IYI1	DMKN	Dermokine	Cornified envelope assembly	10 v 39	1.06	0.002
P01709	IGLV2-8	Immunoglobulin lambda variable 2-8	Antigen recognition	21 v 105	0.35	0.002
P42785	PRCP	Lysosomal Pro-X carboxypeptidase	Carboxypeptidase	7 v 25	0.67	0.002
P31151	S100A7	Protein S100-A7 (Psoriasin)	Inflammatory/immune response	21 v 106	0.97	0.003
A0A494C0G5	AGRN	Agrin	Developmental protein	8 v 60	0.75	0.004
Q8WZ75	ROBO4	Roundabout homolog 4	Developmental protein, angiogenesis	21 v 111	0.43	0.004
P13473	LAMP2	Lysosome-associated membrane glycoprotein 2	Autophagy	20 v 98	0.51	0.006
Q58FF6	HSP90AB4P	Putative heat shock protein HSP 90-beta 4	Molecular chaperone, stress repsonse	15 v 63	1.04	0.009
Q8NDA2	HMCN2	Hemicentin-2	Extracellular matrix	2 v 22	-0.68	0.009
Q92692	NECTIN2	Nectin-2	Modulator of T-cell signalling	15 v 64	0.38	0.012
Q99497	PARK7	Parkinson disease protein 7	Cell protection from oxidative stress	5 v 15	0.66	0.017
P08779	KRT16	Keratin, type I cytoskeletal 16	Cytokeratin	11 v 81	0.50	0.017
Q9UMS6	SYNPO2	Synaptopodin-2	Actin binding, cell migration	21 v 107	-0.49	0.017
P61970	NUTF2	Nuclear transport factor 2	mRNA transport	16 v 87	0.37	0.017
Q15274	QPRT	Nicotinate-nucleotide pyrophosphorylase	Pyridine nucleotide biosynthesis	3 v 11	1.40	0.018
P14780	MMP9	Matrix metalloproteinase-9	Collagen degradation, leukocyte migration	21 v 105	0.30	0.018
Q96RR4	CAMKK2	Calcium/calmodulin-dependent protein kinase kinase 2	Serine/threonine-protein kinase	8 v 42	1.37	0.020
P41181	AQP2	Aquaporin-2	Fluid balance	14 v 70	0.63	0.021
P15289	ARSA	Arylsulfatase A	Lipid metabolism	19 v 76	0.60	0.021
P05543	SERPINA7	Thyroxine-binding globulin	Thyroid hormone transport protein	19 v 96	0.55	0.023
O95865	DDAH2	N(G),N(G)-dimethylarginine dimethylaminohydrolase 2	Regulation of nitric oxide production	2 v 7	0.44	0.023
P31944	CASP14	Caspase-14	Epidermal differentiation	18 v 87	0.46	0.024
Q969E1	LEAP2	Liver-expressed antimicrobial peptide 2	Antimicrobial	18 v 99	1.14	0.024
P29966	MARCKS	Myristoylated alanine-rich C-kinase substrate	Actin/Calmodulin-binding	2 v 8	-0.45	0.025
P25815	S100P	Protein S100-P	Calcium signalling	15 v 85	0.42	0.027
P05091	ALDH2	Aldehyde dehydrogenase, mitochondrial	Oxidoreductase	4 v 26	0.43	0.029
P01019	AGT	Angiotensinogen	Regulation of blood pressure	5 v 28	0.55	0.030
P07998	RNASE1	Ribonuclease pancreatic	Endonuclease	21 v 111	0.32	0.030
Q96HD1	CRELD1	Protein disulfide isomerase	Isomerase	2 v 16	0.55	0.031
P05109	S100A8	Protein S100-A8	Inflammatory/immune response	21 v 107	0.69	0.034
P00738	HP	Haptoglobin	Haemoglobin recycling	21 v 111	0.55	0.035
P39059	COL15A1	Collagen alpha-1(XV) chain	Stabilizes microvessels/muscle cells	14 v 74	0.39	0.035
Q03154	ACY1	Aminoacylase-1	Hydrolase	4 v 18	0.65	0.035
P53634	CTSC	Dipeptidyl peptidase 1 (Cathepsin C)	Thiol protease	19 v 77	0.31	0.038
Q9Y4C0	NRXN3	Neurexin-3	Neuronal cell adhesion/recognition	16 v 50	0.46	0.038
Q96K68	Unknown	cDNA FLJ14473 fis, SNC73 mRNA	Unknown	21 v 111	0.28	0.042
O75882	ATRN	Attractin	Inflammatory response	21 v 97	0.30	0.043
Q9NZH0	GPRC5B	G-protein coupled receptor family C group 5 member B	Unknown	16 v 86	0.37	0.045

P43652	AFM	Afamin	Protein transport	21 v 111	-0.27	0.047
P68032	ACTC1	Actin, alpha cardiac muscle 1	Cytoskeleton	21 v 107	0.24	0.048
P51654	GPC3	Glypican-3	Protease inhibitor	9 v 49	-0.54	0.049

Additional file Table 5: : Significantly altered protein abundances between POLD vs Term-born children

UniProt Accession	Gene Name	Protein Name	Protein Function	POLD vs Term (n = 21 v 46)		
				Replicates	Log_2FC	p
Q9HCU0	CD248	Endosialin	Calcium binding/extra-cellular matrix	21 v 46	0.33	0.0003
O15370	SOX12	Transcription factor SOX-12	Transcription factor	5 v 7	-2.05	0.0003
Q16610	ECM1	Extracellular matrix protein 1	Multifunctional, protease binding	6 v 15	-1.49	0.0004
P69905	HBA1; HBA2	Hemoglobin subunit alpha	Oxygen transport	11 v 37	1.31	0.0004
C9IYI1	DMKN	Dermokine	Cornified envelope assembly	10 v 15	1.21	0.001
Q08380	LGALS3BP	Galectin-3-binding protein	Cell adhesion	21 v 46	-0.58	0.001
Q8WZ75	ROBO4	Roundabout homolog 4	Developmental protein, angiogenesis	21 v 46	0.60	0.001
Q14050	COL9A3	Collagen alpha-3(IX) chain	Extracellular matrix	8 v 7	0.70	0.001
A0A0S2Z4G4	TPM3	Tropomyosin 3 isoform 1 (Fragment)	Actin-binding	2 v 12	0.64	0.002
Q9UNN8	PROCR	Endothelial protein C receptor	Blood coagulation	21 v 46	0.49	0.003
O75594	PGLYRP1	Peptidoglycan recognition protein 1	Innate immunity	21 v 45	0.60	0.003
P98160	HSPG2	Basement membrane-specific heparan sulfate proteoglycan core protein	Basement membrane/angiogenesis	21 v 46	0.28	0.003
P15151	PVR	Poliovirus receptor	Natural killer cell adhesion/activation	14 v 22	0.93	0.004
P40189	IL6ST	Interleukin-6 receptor subunit beta	Cytokine binding	2 v 2	1.30	0.004
P61970	NUTF2	Nuclear transport factor 2	mRNA transport	16 v 41	0.64	0.004
P17936	IGFBP3	Insulin-like growth factor-binding protein 3	Growth regulation	16 v 31	1.39	0.005
U6FVB0	CD74-Ntrk1 fusion gene	Tyrosine-protein kinase receptor	Tyrosine-protein kinase	19 v 39	-0.51	0.005
Q16270	IGFBP7	Insulin-like growth factor-binding protein 7	Growth regulation	21 v 46	-0.80	0.006
P07998	RNASE1	Ribonuclease pancreatic	Endonuclease	21 v 46	0.45	0.008
D3DNU8	KNG1	Kininogen 1, isoform CRA_a	Vasodilation	21 v 46	-0.50	0.008
P09603	CSF1	Macrophage colony-stimulating factor 1	Cytokine	20 v 39	0.46	0.009
P19827	ITIH1	Inter-alpha-trypsin inhibitor heavy chain H1	Serine protease inhibitor	7 v 24	0.78	0.009
P05154	SERPINA5	Plasma serine protease inhibitor	Serine protease inhibitor	19 v 46	-0.52	0.010
P61916	NPC2	NPC intracellular cholesterol transporter 2	Cholesterol metabolism	9 v 23	0.52	0.011
P00738	HP	Haptoglobin	Haemoglobin recycling	21 v 46	0.72	0.011
P07900	HSP90AA1	Heat shock protein HSP 90-alpha	Molecular chaperone	2 v 12	0.71	0.012
P01024	C3	Complement C3	Innate immunity	19 v 46	-0.35	0.012
Q9UQV4	LAMP3	Lysosome-associated membrane glycoprotein 3	Adaptive immunity	15 v 34	-0.49	0.013
Q4TZM4	HBB	Hemoglobin beta chain (Fragment)	Oxygen transport	4 v 7	2.21	0.013
P51654	GPC3	Glypican-3	Protease inhibitor	9 v 24	-0.77	0.013
Q8NDA2	HMCN2	Hemicentin-2	Extracellular matrix	2 v 9	-0.65	0.013
Q96S96	PEBP4	Phosphatidylethanolamine-binding protein 4	Extracellular exosome	12 v 18	0.74	0.013
A0A494C0G5	AGRN	Agrin	Developmental protein	8 v 5	0.62	0.013
Q14982	OPCML	Opioid-binding protein/cell adhesion molecule	Cell adhesion/opioid binding	15 v 45	-0.35	0.014

P01019	AGT	Angiotensinogen	Regulation of blood pressure	5 v 8	1.02	0.014
Q99985	SEMA3C	Semaphorin-3C	Developmental protein	8 v 14	2.13	0.015
P13473	LAMP2	Lysosome-associated membrane glycoprotein 2	Autophagy	20 v 46	0.50	0.015
P19652	ORM2	Alpha-1-acid glycoprotein 2	Transport protein	23 v 46	-0.46	0.016
P08779	KRT16	Keratin, type I cytoskeletal 16	Cytokeratin	11 v 38	0.52	0.017
P19835	CEL	Bile salt-activated lipase	Serine esterase, lipid degredation	15 v 22	0.57	0.018
O43866	CD5L	CD5 antigen-like (Apoptosis inhibitor expressed by macrophages)	Inflammatory response regulation	8 v 25	-0.79	0.018
P42785	PRCP	Lysosomal Pro-X carboxypeptidase	Carboxypeptidase	7 v 28	0.46	0.019
P41181	AQP2	Aquaporin-2	Fluid balance	14 v 46	0.69	0.020
P39059	COL15A1	Collagen alpha-1(XV) chain	Stabilizes microvessels/muscle cells	14 v 24	0.46	0.020
P0DOX7	Unknown	Immunoglobulin kappa light chain	Immune response	7 v 17	0.55	0.020
P52848	NDST1	Bifunctional heparan sulfate N-deacetylase/N-sulfotransferase 1	Multifunctional enzyme/immune response	21 v 44	-0.92	0.021
O15240	VGF	Neurosecretory protein VGF	Neurogenesis/neuroplasticity	21 v 46	-0.49	0.021
Q96K68	Unknown	cDNA FLJ14473 fis	Cell membrane	21 v 46	0.33	0.021
P00749	PLAU	Urokinase-type plasminogen activator	Plasminogen activator	13 v 31	-0.53	0.022
A0A193CHQ9	Unknown	10E8 heavy chain variable region (Fragment)	Unknown	19 v 43	0.53	0.023
Q9UMX5	NENF	Neudesin	Neurotrophic factor	14 v 21	0.48	0.025
A8TX70	COL6A5	Collagen alpha-5(VI) chain	Cell-binding protein	16 v 28	-0.52	0.025
P15144	ANPEP	Aminopeptidase N	Aminopeptidase	20 v 38	0.39	0.025
A0A140T9A1	COL11A2	Collagen alpha-2(XI) chain	Extracellular matrix	20 v 36	-0.55	0.029
Q6UVK1	CSPG4	Chondroitin sulfate proteoglycan 4	Cell proliferation/migration	21 v 46	0.58	0.031
P78380	OLR1	Oxidized low-density lipoprotein receptor 1	Inflammatory response	4 v 18	-0.44	0.033
P29508	SERPINB3	Serpin B3	Serine protease inhibitor	11 v 25	0.61	0.036
P98095	FBLN2	Fibulin-2	Binds to fibronectin	21 v 46	0.31	0.039
Q9NZH0	GPRC5B	G-protein coupled receptor family C group 5 member B	Unknown	16 v 34	0.43	0.039
Q9NRX4	PHPT1	14 kDa phosphohistidine phosphatase	Protein phosphatase	7 v 11	0.89	0.039
A0A2U8J953	IgH	Ig heavy chain variable region (Fragment)	Immune response	6 v 7	-1.25	0.041
P43121	MCAM	Cell surface glycoprotein MUC18	Cell adhesion	21 v 46	0.45	0.044
P02774	GC	Vitamin D-binding protein	Vitamin D transport/storage	8 v 14	-1.17	0.045
P01133	EGF	Pro-epidermal growth factor	Growth factor	21 v 46	-0.42	0.045
P01709	IGLV2-8	Immunoglobulin lambda variable 2-8	Antigen recognition	21 v 46	0.30	0.045
P33908	MAN1A1	Mannosyl-oligosaccharide 1,2-alpha-mannosidase IA	Glycosidase/hydrolase	18 v 35	-0.39	0.045
P02766	TTR	Transthyretin	Thyroid hormone-binding protein	21 v 41	0.50	0.046
P14780	MMP9	Matrix metalloproteinase-9	Collagen degradation, leukocyte migration	21 v 46	0.26	0.046
Q12860	CNTN1	Contactin-1	Cell adhesion/signalling	17 v 45	-0.36	0.048
Q8NEJ1	Unknown	Immunoglobulin lambda-1 light chain-like	Cell membrane	3 v 7	-1.05	0.048

Additional file Table 6: ROC Analysis (using Leave-One-Out Cross Validation model) of high replicate proteins implicated in related biological functions by IPA software. POLD: Prematurity-related obstructive lung disease. pPRISm: Prematurity-related preserved ratio with impaired spirometry. PT_c: Preterm-born controls. PPV: Positive predictive value. NPV: Negative predictive value

Protein(s) included in model	Replicates	AUC (95% CI)	p-value	Sensitivity (95% CI)	Specificity (95% CI)	PPV (95% CI)	NPV (95% CI)
**pPRISm vs PT_c (n=27 v 112)**							
**DNASE1**	27 v 112	0.62 (0.51, 0.74)	0.024	0.56 (0.37, 0.74)	0.69 (0.61, 0.78)	0.31 (0.18, 0.44)	0.87 (0.79, 0.94)
**PGLYRP1**	27 v 112	0.61 (0.53, 0.70)	0.005	0.54 (0.40, 0.67)	0.67 (0.61, 0.73)	0.28 (0.20, 0.37)	0.86 (0.80, 0.91)
**B2M**	27 v 112	0.58 (0.46, 0.71)	0.91	0.59 (0.41, 0.78)	0.64 (0.55, 0.73)	0.29 (0.17, 0.40)	0.87 (0.79, 0.94)
**SERPINA3**	25 v 100	0.63 (0.49, 0.76)	0.97	0.48 (0.28, 0.68)	0.80 (0.72, 0.88)	0.38 (0.21, 0.54)	0.86 (0.79, 0.93)
**DNASE1 + PGLYRP1 + B2M + SERPINA3**	25 v 100	0.65 (0.52, 0.78)	0.010	0.76 (0.59, 0.93)	0.66 (0.57, 0.75)	0.36 (0.23, 0.49)	0.92 (0.85, 0.98)
**POLD vs PT_c (n=21 v 112)**							
**S100A8**	21 v 107	0.59 (0.47, 0.72)	0.92	0.82 (0.66, 0.98)	0.45 (0.35, 0.54)	0.23 (0.14, 0.33)	0.92 (0.85, 0.99)
**MMP9**	21 v 105	0.59 (0.45, 0.73)	0.90	0.38 (0.17, 0.59)	0.84 (0.77, 0.91)	0.32 (0.14, 0.50)	0.87 (0.81, 0.94)
**CTSC**	19 v 77	0.59 (0.46, 0.73)	0.90	0.58 (0.36, 0.80)	0.66 (0.56, 0.77)	0.30 (0.15, 0.45)	0.86 (0.78, 0.95)
**S100A8 + MMP9 + CTSC**	19 v 77	0.72 (0.57, 0.86)	0.002	0.47 (0.25, 0.70)	0.91 (0.85, 0.97)	0.56 (0.32, 0.81)	0.88 (0.80, 0.95)

Additional file Figure 3: ROC Curves (using Leave-One-Out Cross Validation model) of protein groups with highest AUC values (A) pPRISm vs PT_c including DNASE1, PGLYRP1, B2M and SERPINA3 (B) POLD vs PT_c including S100A8, MMP9 and CTSC. POLD: Prematurity-related obstructive lung disease. pPRISm: Prematurity-related preserved ratio with impaired spirometry. PT_c: Preterm-born controls

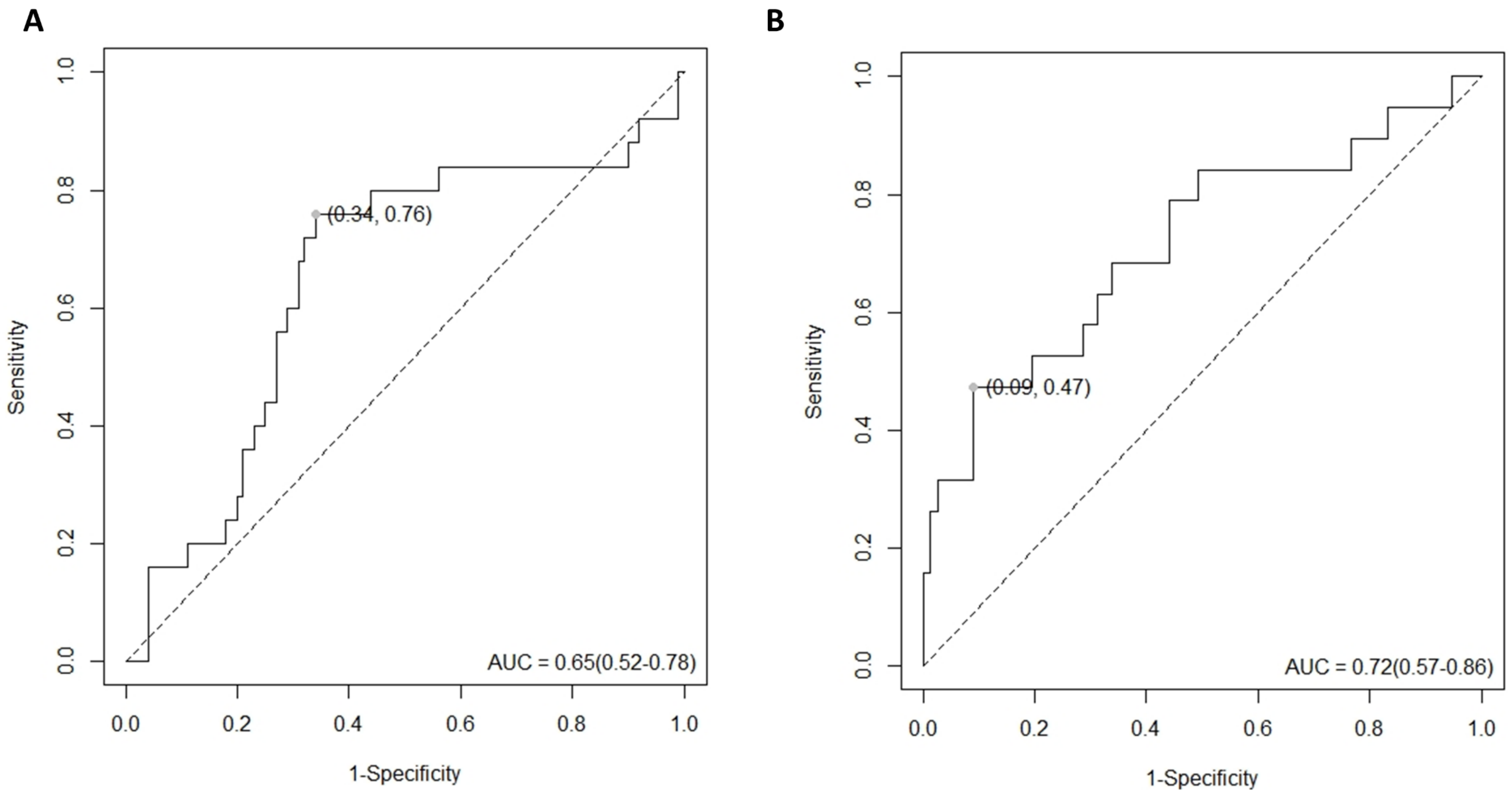

Supplement: Supplementary file 1 — Additional file 1: Contains supplementary figures and tables to give further detail to the methods and results section [file 12931_2023_2494_MOESM1_ESM.pdf]
